# Vestibular agnosia in traumatic brain injury and its link to imbalance

**DOI:** 10.1093/brain/awaa386

**Published:** 2020-12-26

**Authors:** Elena Calzolari, Mariya Chepisheva, Rebecca M Smith, Mohammad Mahmud, Peter J Hellyer, Vassilios Tahtis, Qadeer Arshad, Amy Jolly, Mark Wilson, Heiko Rust, David J Sharp, Barry M Seemungal

**Affiliations:** 1 Brain and Vestibular Group, Neuro-Otology Unit, Department of Brain Sciences, Charing Cross Hospital, Imperial College London, London, W6 8RF, UK; 2 Centre for Neuroimaging Sciences, King’s College London, London WC2R 2LS, UK; 3 King’s College Hospital NHS Foundation Trust, SE5 9RS, UK; 4 InAmind Laboratory, Department of Neuroscience, Psychology and Behaviour, University of Leicester, Leicester, LE1 7RH, UK; 5 C3NL, Department of Brain Sciences, Hammersmith Hospital, Imperial College London, London, W12 0NN, UK; 6 St Mary’s Hospital Major Trauma Centre, Imperial College Healthcare NHS Trust, London, W2 1NY, UK

**Keywords:** vestibular agnosia, traumatic brain injury, self-motion perception, vestibular cognition, vertigo

## Abstract

Vestibular dysfunction, causing dizziness and imbalance, is a common yet poorly understood feature in patients with TBI. Damage to the inner ear, nerve, brainstem, cerebellum and cerebral hemispheres may all affect vestibular functioning, hence, a multi-level assessment—from reflex to perception—is required. In a previous report, postural instability was the commonest neurological feature in ambulating acute patients with TBI. During ward assessment, we also frequently observe a loss of vertigo sensation in patients with acute TBI, common inner ear conditions and a related vigorous vestibular-ocular reflex nystagmus, suggesting a ‘vestibular agnosia’. Patients with vestibular agnosia were also more unbalanced; however, the link between vestibular agnosia and imbalance was confounded by the presence of inner ear conditions. We investigated the brain mechanisms of imbalance in acute TBI, its link with vestibular agnosia, and potential clinical impact, by prospective laboratory assessment of vestibular function, from reflex to perception, in patients with preserved peripheral vestibular function. Assessment included: vestibular reflex function, vestibular perception by participants’ report of their passive yaw rotations in the dark, objective balance via posturography, subjective symptoms via questionnaires, and structural neuroimaging. We prospectively screened 918 acute admissions, assessed 146 and recruited 37. Compared to 37 matched controls, patients showed elevated vestibular-perceptual thresholds (patients 12.92°/s versus 3.87°/s) but normal vestibular-ocular reflex thresholds (patients 2.52°/s versus 1.78°/s). Patients with elevated vestibular-perceptual thresholds [3 standard deviations (SD) above controls’ average], were designated as having vestibular agnosia, and displayed worse posturography than non-vestibular-agnosia patients, despite no difference in vestibular symptom scores. Only in patients with impaired postural control (3 SD above controls’ mean), whole brain diffusion tensor voxel-wise analysis showed elevated mean diffusivity (and trend lower fractional anisotropy) in the inferior longitudinal fasciculus in the right temporal lobe that correlated with vestibular agnosia severity. Thus, impaired balance and vestibular agnosia are co-localized to the inferior longitudinal fasciculus in the right temporal lobe. Finally, a clinical audit showed a sevenfold reduction in clinician recognition of a common peripheral vestibular condition (benign paroxysmal positional vertigo) in acute patients with clinically apparent vestibular agnosia. That vestibular agnosia patients show worse balance, but without increased dizziness symptoms, explains why clinicians may miss treatable vestibular diagnoses in these patients. In conclusion, vestibular agnosia mediates imbalance in traumatic brain injury both directly via white matter tract damage in the right temporal lobe, and indirectly via reduced clinical recognition of common, treatable vestibular diagnoses.

## Teaser

Calzolari *et al.* show that acute traumatic brain injury may cause a ‘vestibular agnosia’. This mediates imbalance both directly via white matter tract damage in the right temporal lobe, and indirectly by reducing clinician recognition of common, treatable vestibular diagnoses.

## Introduction

Traumatic brain injury (TBI) is the commonest cause of chronic disability in young adults (Langlois *et al.*, 2006) and imbalance post-TBI is a key predictor of failure to return to work (Chamelian and Feinstein, 2004; Maskell *et al.*, 2006). Indeed, even in mild TBI patients, return-to-work rates at 6 months are reduced from 75% in those without vestibular features, to 33% in those with vestibular dysfunction (Chamelian and Feinstein, 2004). TBI is an independent predictor of falls (Maskell *et al.*, 2006, 2007), even in young adults, perhaps explaining why imbalance in TBI also predicts return-to-work rates. Despite its importance in recovery from TBI, the mechanisms underlying chronic post-TBI imbalance and vestibular functioning in general are poorly understood, with one large study unable to identify any specific cause in 25% of chronic TBI patients (Hoffer *et al.*, 2007).

One reason for a relative lack of understanding of imbalance in TBI is that there are no acute prospective studies (mechanistic or otherwise) assessing vestibular dysfunction in acute TBI. There has been one cross-sectional study assessing instrumented measures of balance, in the subacute stage (Klima *et al.*, 2019) confirming manifest imbalance in subacute TBI. We also recently reported two separate cross-sectional clinical studies in ambulant acute TBI patients; one showing that 62% were unbalanced (Marcus *et al.*, 2019) of whom half did not report feeling unbalanced, and the other (Sargeant *et al.*, 2018), confirming the lack of correlation between objective signs of imbalance and vestibular symptom scores.

Another common diagnosis in acute TBI patients is benign paroxysmal positional vertigo (BPPV), affecting ∼40% of cases in our acute cross-sectional series (Marcus *et al.*, 2019). Consistent with our documented observation of a dissociation between objective and subjective features of vestibular dysfunction in acute TBI (Sargeant *et al.*, 2018; Marcus *et al.*, 2019), we noted several acute TBI patients who denied vertigo sensation despite an obvious vestibular ocular reflex (VOR) response (e.g. during the positional manoeuvre used to diagnose BPPV), i.e. a loss of the vestibular perception of self-motion or ‘vestibular agnosia’ (Supplementary Video 1). Our empirical observation in acute TBI patients on the major trauma ward suggested that those patients with a clinically apparent vestibular agnosia (i.e. a vestibular agnosia that is sufficiently severe to be visible on bedside testing as seen in the above clinical video), were also those with worse balance function. This observation was confounded, however, by the clinical situation where the stimulus to the peripheral vestibular apparatus required to reveal both a prominent nystagmus and simultaneous lack of vertigo sensation, could only occur in the setting of a co-existing inner ear disorder, such as BPPV (or an acute vestibular nerve injury that affected 19% of acute TBI cases; Marcus *et al.*, 2019). In inner ear conditions, in addition to a reflex vestibular nystagmus, patients with a healthy brain (i.e. not acute TBI cases), complain of severe vertigo. To assess whether vestibular agnosia was directly linked to imbalance required the formal testing of vestibular reflex and perceptual function in acute TBI patients in whom there was no inner ear dysfunction, along with laboratory assessment of balance function. Our general hypothesis linking vestibular agnosia and imbalance was simply that reduced vestibular signalling at cerebral cortical level would manifest both in a vestibular agnosia as well as imbalance (given the cortical dominance of postural control in humans). An additional question was whether there are cortical regions that colocalise the functions of vestibular perception and vestibular-mediated balance control, which is of potential interest to the understanding of the brain’s control of balance, since balance control mechanisms in humans are poorly understood, particularly compared to that in quadrupeds.

The bedside observation of a loss of vertigo sensation in patients with preserved inner ear functioning (vestibular agnosia) has received scant attention but has hitherto been only empirically reported in elderly patients, typically with cerebral small vessel disease (Seemungal, 2005; Imbaud Genieys, 2007; Chiarovano *et al.*, 2016). Conversely, prospective laboratory assessment in acute (within 2 weeks), focal stroke patients (Kaski *et al.*, 2016), found no evidence of a vestibular agnosia. The mechanism linking vestibular agnosia with elderly small vessel disease and young patients with acute TBI may relate to the hypothesis that the vestibular sensation of self-motion is mediated by a distributed cortical network (Seemungal, 2014; Nigmatullina *et al.*, 2015) that becomes disconnected, potentially explaining why acute TBI cases may be susceptible to vestibular agnosia since this patient group exhibit cognitive deficits that often relate to the disruption of cortical networks (Ham and Sharp, 2012).

Given our aim to link vestibular agnosia with imbalance both behaviourally and by neuroanatomical substrate, we therefore prospectively screened patients admitted to a major trauma ward eligible for recruitment, and in whom we assessed: (i) VOR and vestibular-perceptual thresholds (Seemungal *et al.*, 2004); (ii) posturography; and (iii) patients’ whole brain white matter microstructure with diffusion tensor imaging (DTI) via fractional anisotropy (FA) and mean diffusivity (MD), and then correlated DTI parameters with our behavioural parameters of interest (vestibular-motion perceptual thresholds, and posturography).

Finally, given our prospective screening of all acute TBI cases presenting to the major trauma ward, we could also assess whether clinically apparent vestibular agnosia affected referral patterns to our clinical ‘dizzy’ service from clinicians managing acute TBI patients. Hence a secondary more clinically related hypothesis was that clinically apparent vestibular agnosia masks symptoms and hence reduces the likelihood of patients with vestibular diagnoses being referred for treatment. Thus, untreated inner ear disorders could explain, at least in part, the empirical observation of worse balance in vestibular agnosia cases.

Thus using a combination of laboratory testing and comprehensive clinical screening facilitating clinical audit of acute TBI cases, we assessed our hypotheses that imbalance in acute TBI is due to: (i) specific damage to cortical circuits that mediate imbalance may show neuroanatomical overlap with circuits mediating vestibular agnosia; and (ii) an indirect effect of vestibular agnosia leading to the loss of clinical recognition, and hence a failure to treat common vestibular diagnoses in acute TBI cases.

## Materials and methods

### Participants and recruitment

Patients admitted to the St Mary’s Hospital Major Trauma Centre (London, UK) were systematically screened twice weekly via consultant-led ward rounds for inclusion into our study. Importantly, the criteria for inclusion did not include complaints of vestibular dysfunction, but the main inclusion criterion (see below for the complete inclusion/exclusion criteria) was that the patient had sustained a blunt head injury requiring admission to the major trauma ward. Patients were also assessed following referral by admitting clinicians (neurosurgery, emergency medicine and neuro-physiotherapy) for imbalance and/or dizziness. Patient recruitment occurred between August 2017 and January 2020, with the first 9 months clinically audited. Acute TBI patients were also recruited from King’s College Hospital in the final 6 months. Patients without capacity were recruited via a consultee and informed patient consent obtained at a subsequent follow-up. Before testing, and typically whilst patients were on the ward, any BPPV was treated by repositioning manoeuvres, and residual migraine-phenotype headaches treated medically with 3–5 days of naproxen and prochlorperazine. Hence, on the day of laboratory testing, patients were free of active vestibular problems due to BPPV or migraine.

Inclusion criteria were: (i) blunt head injury resulting in admission to the major trauma ward; (ii) age 18–65; and (iii) preserved peripheral vestibular function. Exclusion criteria were: (i) additional active pre-morbid medical, neurological, or psychiatric condition (unless inactive or controlled); (ii) musculoskeletal condition impairing ability to balance; (iii) substance abuse history; (iv) pregnancy; and (v) inability to obtain consent or assent.

Thirty-seven matched healthy controls were recruited following written informed consent. The study was conducted in accordance with the principles of the Declaration of Helsinki and was approved by the local Research Ethics Committee.

### Procedure

All participants completed assessment of peripheral and reflex vestibular function, vestibular perceptual testing, reaction times, posturography, and neuroimaging. Patients additionally were assessed with symptom questionnaires assessing perceived dizziness and imbalance, and a cognitive battery (Addenbrooke's Cognitive Examination Revised, ACE-R) (Table 1).

**Table 1:** Demographic and clinical details of patients tested

Pt	Gender: Age	GCS	MOI	Days from injury	PTA, days	Severity MAYO	CT brain lesions	Skull fracture	DAI on MRI	BPPV	ACE-R
01	M : 65	12	RTA	9	9	Mod-Sev	R, L SDH, SAH	+	−	+	82
02	M : 22	14	RTA	7	7	Mod-Sev	R, L SDH	+	−	+	77
03	M : 49	14	Fall	14	6^b^	Mod-Sev	R, L SDH, SAH	+	+	+	80
04	F : 48	15	RTA	2	0	Mod-Sev	L SAH	−	−	−	77
05	F : 41	14	RTA	3	0	Mild-Prob	None	−	−	+	97
06	M : 40	13	Fall	20	20	Mod-Sev	R, L SDH, SAH	+	−	−	51
07	F : 43	15	RTA	12	0	Mild-Prob	No deficit	−	−	−	100
08	M : 23	13	Assault	10	0	Mod-Sev	R, L SDH, SAH	+	+	−	78
09	F : 54	15	RTA	5	0	Mod-Sev	R SDH	−	−	−	90
10	F : 59	14	Fall	2	0	Mod-Sev	R, L SDH	−	+	+	76
11	M : 65	14	Fall	21	13	Mod-Sev	R SDH, L SAH	+	−	−	76
12	M : 37	14	RTA	11	20	Mod-Sev	None	+	+	−	67
13	F : 62	15	Fall	13	0	Mild-Prob	None	−	−	+	99
14	M : 40	15	Fall	4	0	Mod-Sev	R SAH	+	−	+	96
15	M : 35	15	RTA	15	0	Mod-Sev	R, L SAH	+	+	+	86
16	M : 26	15	Fall	2	0	Mod-Sev	None	+	−	−	83
17	M : 58	15	Fall	6	0	Mod-Sev	L SAH	−	+	+	90
18	M : 42	15	RTA	7	0	Mod-Sev	R, L SDH, SAH	+	+	−	86
19	M : 30	15	Fall	11	0	Mod-Sev	None	−	−	+	100
20	M : 43	14	Fall	17	0	Mod-Sev	R, L SAH	+	−	−	94
21	F : 60	15	Fall	4	0	Mod-Sev	R, L SDH, SAH	+	−	−	96
22	M : 47	15	Fall	12	0	Mod-Sev	L SDH, SAH	−	−	−	98
23	M : 49	14	Fall	19	10	Mod-Sev	R, L SDH, SAH	+	−	+	93
24	M : 41	13	RTA	19	20	Mod-Sev	R, L SDH, SAH	−	−	−	70
25	M : 47	15	Fall	15	12	Mod-Sev	R, L SDH	+	−	+	95
26	F : 34	14	Fall	34	31	Mod-Sev	R, L SDH, SAH	+	−	+	69
27	M : 24	8	Assault	20	9	Mod-Sev	R, L SDH, SAH	+	+	−	93
28	F : 33	14	RTA	10	12	Mod-Sev	R SDH, SAH	−	−	−	65
29	F : 40	13	Fall	33	0	Mod-Sev	L SDH, SAH	+	+	−	90
30	M : 56	14	Fall	15	0	Mod-Sev	R, L SDH	−	−	+	94
31	M : 39	15	Fall	29	0	Mod-Sev	R, L SDH, SAH	+	+	+	99
32	M : 18	15	RTA	16	9^b^	Mod-Sev	None	−	+	−	77
33	M : 34	3^a^	Assault	28	24^b^	Mod-Sev	R, L SDH	+	−	−	89
34	M : 48	14	Fall	14	14^b^	Mod-Sev	R, L SDH, SAH	+	−	+	100
35	M : 59	15	Fall	22	0	Mild-Prob	None	−	+	+	90
36	M : 36	12	RTA	77	61^b^	Mod-Sev	L SDH, SAH	+	−	+	np
37	F : 20	15	RTA	24	12	Mod-Sev	R, L SDH, SAH	+	+	−	84

+/− = present/absent; ACE-R = The Addenbrooke's Cognitive Examination Revised; DAI = diffuse axonal injury; GCS = Glasgow Coma Scale (in Accident and Emergency); L = left; Mod/Sev/Prob = moderate/severe/probable; MOI = mechanism of injury; np = not performed; PTA = post traumatic amnesia; R = right; RTA = road traffic accident; SAH = subarachnoid haemorrhage; SDH = subdural haemorrhage.

a Actual GSC unattainable as the patient was intubated at scene.

b Estimated minimum.

### Clinical assessment on the acute ward

The clinical assessment of a patient with an acute TBI included a general physical and neurological examination, including a cognitive assessment and the patient’s capacity to understand the process of informed consent. We also assessed patients for signs of peripheral vestibular loss. This included: (i) the doll’s eyes and head impulse manoeuvre; (ii) assessing for nystagmus in the primary position including via fundoscopy both with an without visual fixation; and (iii) fundoscopic examination whilst passively oscillating the head at 2 Hz to assess for the preservation of the VOR in right- and leftward directions. Clinical assessment also included a general oculomotor exam and gait assessment including assessing for the Romberg sign, tandem standing with eyes open and closed, each for 20 s and, checking for the number of mistakes during 10 tandem steps.

### Laboratory assessment of peripheral and reflex vestibular function

To exclude end-organ dysfunction as a cause for impaired vestibular perception, all participants had VOR assessment (Table 2). Testing included video head impulse testing and rotational chair testing with eye movement assessment of VOR gain for the stopping response from 90°/s constant rotation. We also recorded VOR gain for whole body oscillations in the dark between 0.1 Hz to 0.4 Hz (not reported here). The laboratory measures (Table 2) were within the normal limits for all patients included in the study.

**Table 2:** Peripheral and reflex vestibular function of patients

	vHIT			Caloric		90°/s rotation gain			
Patient	Asymmetry	L gain	R gain	RC SPV	LC SPV	R - rot	L - rot	R - stop	L- stop
01^a^	4%^b^	1.29	1.21	–	–	0.94	0.96	0.64	0.60
02^a^	6%^b^	0.96	1.09	–	–	0.57	0.56	0.45	0.43
03^a^	–	–	–	64°/s	−80°/s	1.02	1.02	0.58	0.40
04^a^	4%^b^^,c^	0.84	0.91	–	–	0.61^c^	0.62^c^	0.47^c^	0.50^c^
05	–	–	–	–	–	0.71	0.58	0.51	0.50
06^a^	3%^b^^,c^	1.02	1.09	–	–	1.01^c^	0.72^c^	0.48^c^	0.50^c^
07	4%^d^	0.89	0.93	–	–	0.74	0.78	0.54	0.48
08^a^	2%^d^	0.91	0.93	–	–		
09	1%^d^	0.97	0.98	–	–	0.66	0.71	0.52	0.61
10^a^	4%^d^	1.00	0.96	–	–	0.76	0.61	0.47	0.54
11^a^	2%^b^	0.78	0.81	–	–	0.78	0.61	0.58	0.55
12^a^	13%^d^	0.82	0.71	–	–	0.64^c^	0.63^c^	0.60^c^	0.63^c^
13	3%^d^	1.15	1.18	–	–	0.59	0.55	0.51	0.48
14	–	–	–	47°/s	−38°/s	0.55	0.54	0.54	0.54
15	1%^b^^,e^	1.11	1.09	–	–	0.70^e^	0.53^e^	0.45^e^	0.44^e^
16	3%^b^	1.27	1.34	–	–	0.77	0.62	0.61	0.62
17	2%^b^	1.23	1.28	–	–	1.02	0.91	1.08	1.08
18^a^	2%^b^	0.88	0.92	–	–	0.98	0.86	0.82	0.79
19	–	–	–	–	–	0.66	0.67	0.65	0.67
20^a^	5%^b^	0.98	1.09	–	–	0.89	0.71	0.72	0.68
21	6%^b^	1.10	0.90	–	–	0.63	0.58	0.54	0.52
22	6%^b^	0.89	1.00	–	–	0.71	0.59	0.55	0.64
23^a^	3%^b^	1.14	1.07	–	–	0.81	0.88	0.68	0.61
24^a^	3%^b^	0.97	1.03	–	–	0.66^e^	0.80^e^	0.44^e^	0.38^e^
25	6%^b^	1.11	0.98	–	–	0.75	0.67	0.58	0.53
26	–	–	–	–	–	0.71	0.85	0.83	0.74
27	7%^b^	1.44	1.25	–	–	0.38	0.66	0.30	0.29
28	4%^b^	0.96	1.04	–	–	0.74	0.72	0.41	0.45
29	10%^b^	1.32	1.08	–	–	0.27	0.34	0.31	0.36
30	7%^b^	0.93	1.06	–	–	0.55	0.53	0.50	0.40
31	7%^b^	0.84	0.96	–	–	0.63	0.63	0.66	0.67
32	9%^b^	1.12	0.93	–	–	0.88	0.90	0.64	0.69
33^a^	2%^b^	1.05	1.10	–	–	0.63	0.79	0.57	0.75
34	6%^b^	0.98	1.10	–	–	0.87	0.86	0.82	0.82
35	2%^b^	1.07	1.12	–	–	0.75	0.80	0.71	0.64
36^a^	3%^b^	1.31	1.23	–	–	0.98	0.92	0.86	0.87
37	–	–	–	–	–	1.01	0.89	0.76	0.76

To rule out the presence of peripheral vestibular deficit, participants underwent VOR assessment via video head impulse test, or via other VOR assessment techniques appropriate for the patient’s clinical findings (e.g. bithermal caloric irrigation or electronystagmography with rotational chair testing was performed if a video head impulse test was not possible due to neck injury). The assessment was performed according to the standard clinical procedures of the Vestibular Neurology laboratory (Charing Cross Hospital, Imperial College London NHS Trust). L = left; LC = left canal; R = right; RC = right canal; SPV = slow phase velocity; vHIT = video head impulse test. A dash indicates where the test was not completed, or adequate recordings obtained.

a Elevated vestibular perceptual thresholds.

b Interacoustic.

c Examination performed at follow-up 3 months.

d Otometrics.

e Examination performed at follow-up 6 months.

### Vestibular perceptual and vestibular ocular reflex thresholds

**Figure 1:**
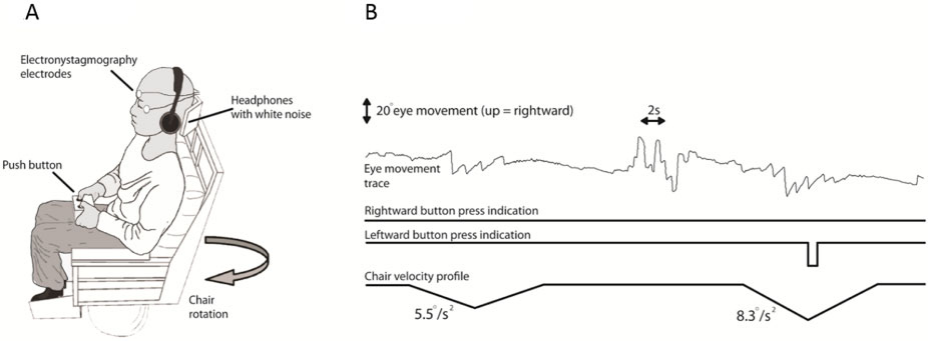
**Vestibular thresholds.** Apparatus and methods. (**A**) Participants sat on a computer-controlled rotating chair (earth-vertical axis). Horizontal eye movements were recorded with electro-nystagmography. Participants indicated their perceived direction of motion by pressing a button to indicate leftward or rightward motion. White noise was delivered through earphones. (**B**) Raw traces for two subsequent rotations for Patient 08. The *top* trace shows the electro-nystagmography signal. The *middle* and *bottom* traces show the patient’s button press to indicate perceived motion direction, right and left. In this example, the chair rotated from rest to the left, at constant acceleration. The chair continues to accelerate until a correct button response is made or if 5 s has elapsed without a correct button press, or no button press, as here. As no response was made during the test period over 5 s, the chair underwent a controlled deceleration to a stop over 5 s. The second rotation, here also to the left (the rotation directions are randomized), was of increased angular acceleration as determined by the Modified Binary Search (MOBS) algorithm (Tyrrell and Owens, 1988; see Supplementary material for further details). In general, for a given direction (left versus right), a non-perceived rotation is followed by a rotation of higher acceleration, while a perceived rotation is followed by a lower acceleration rotation. Further detail on how the test terminates, and hence thresholds obtained for left and right rotations, can be found in the Supplementary material.

Figure 1 shows the apparatus and method used to objectively quantify vestibular perception of self-motion during passive, yaw-plane, whole-body rotations in darkness (Seemungal *et al.*, 2004). This task requires: (i) preserved peripheral vestibular function indicated by evoked VOR nystagmus whose threshold is indicated by the angular velocity at which it was first observed; and (ii) the cognitive ability to perceive this vestibular-mediated signal of self-motion (Seemungal *et al.*, 2004). Participants sat on a rotating chair in total darkness and were instructed to press a button (right or left) as soon as they perceived their movement and its direction (Fig. 1A and B). After each rotation, the lights were turned on to allow post-rotatory vestibular effects to decay. White noise via earphones masked auditory cues. All participants were also tested with a modified threshold technique to ensure consistent results for VOR thresholds as explained further in the Supplementary material.

### Reaction time and vigilance

Slowed reaction times and impaired vigilance, common after TBI (Bonnelle *et al.*, 2011), could result in a spuriously elevated vestibular perceptual threshold. Therefore, all participants performed a visual reaction time task to control for this confound. Participants were required to press a ‘right’ or ‘left’ button as quickly as possible and in congruent fashion, to a right or left pointing arrow that appeared on a PC screen (see Supplementary material for details). Mean reaction times were measured from stimulus onset to the time of a correct button press.

### Posturography

Postural sway using a force platform (further details in the Supplementary material) was measured under four conditions each of 60-s duration: hard surface with eyes open, hard surface with eyes closed, soft surface with eyes open and soft surface with eyes closed, in counterbalanced order (Latin square). Participants stood on a soft rectangular foam pad (50 × 41 × 6 cm, Airex^®^) for the soft conditions. Participants were required to stand with their arms hanging loosely by their sides, and heels 8-cm apart, and told to maintain their balance as best they could. For security, participants wore a chest harness secured to the ceiling. The area of the 95% bivariate confidence ellipse, which encloses 95% of the points on the centre of pressure path along the anteroposterior and mediolateral planes combined, was calculated for each condition, and expressed in square millimetres. The four conditions varied in sensory feedback. In the hard surface-eyes open condition, visual, proprioceptive and vestibular input were available for use in postural control. In the hard surface-eyes closed condition, only proprioception and vestibular input was available. The soft surface conditions impaired proprioceptive input, thus the soft surface-eyes open condition, used primarily visual and vestibular input, and the soft surface-eyes closed condition was primarily vestibular-dependent.

### Dizziness Handicap Inventory and Activity-Specific Balance Confidence Scale

Acute TBI patients completed two questionnaires estimating their perceived disability caused by vertigo sensations [Dizziness Handicap Inventory (DHI); Jacobson and Newman, 1990], and confidence in their balance [Activity-Specific Balance Confidence Scale (ABC); Powell and Myers, 1995].

### Clinical audit of benign paroxysmal positional vertigo and patient referral source

From the initiation of the study in August 2017 to May 2018 (a 9-month period) we prospectively audited the presence or absence of BPPV in the patients we assessed. We noted whether the patient had been referred to our clinical vestibular referral service by the therapy team, or if the BPPV was picked up by our screening approach. We asked patients if they felt dizzy during a BPPV-evoked positional nystagmus. We specifically asked: Do you feel dizzy, yes or no? If yes, is it a severe, moderate or mild feeling of movement? Patients who answered no to ‘are you dizzy?’ were classified as having a clinically apparent vestibular agnosia.

### Statistical analysis

#### Vestibular-perceptual and vestibular-ocular thresholds

A repeated-measures ANOVA tested the differences between patients and healthy controls for vestibular-ocular versus vestibular-perceptual thresholds, expressed in angular velocity (°/s). Patients were classified as having vestibular agnosia if their vestibular-perceptual thresholds were 3 standard deviations (SD) above the controls’ average.

#### Reaction times

Reaction time differences (s) of patients versus controls were tested using univariate ANOVA, reaction times of patients with and without vestibular agnosia were tested using an independent sample *t-*test.

#### Posturography

Differences in sway area (see above for detail) expressed in square millimetres, in the four conditions, between acute TBI with vestibular agnosia, without vestibular agnosia and controls were tested with a repeated measures ANOVA. Patients were classified as balance impaired if their average sway in the vestibular-mediated condition (soft surface-eyes closed) was 3 SD above the controls’ average.

#### Dizziness Handicap Inventory and Activity-Specific Balance Confidence Scale

Differences in dizziness symptoms between TBI patients with and without vestibular agnosia were tested via independent sample *t*-test. Linear correlations assessed whether dizziness symptom scores (DHI) predicted vestibular-perceptual thresholds and whether the balance confidence (ABC) score predicted objective balance via posturography. Differences in balance confidence between groups were tested via independent sample *t*-test.

For all analyses, the alpha-level was set at 0.05. Differences in the means for the significant effects and interactions were explored with Bonferroni *post hoc* correction. To quantify the magnitude of the effects we report, we provide partial eta squared (η_p_^2^) values for *F*-tests.

### Neuroimaging

In a previous study we found that acute, unilateral hemispheric stroke did not cause vestibular agnosia (Kaski *et al.*, 2016). In our current acute TBI cohort, some of the patients with severe vestibular agnosia had no observable contusions on structural neuroimaging on admission (CT and MRI; see the Supplementary material for link to all images), obviating a lesion-mapping approach. We therefore investigated differences in white matter microstructure in a group of acute patients with TBI and an age-matched control group.

#### White matter structural imaging

DTI sequences were acquired on a 3 T Siemens Verio (Siemens) scanner, using a 32-channel head coil. Diffusion-weighted volumes were acquired using a 64-direction protocol: 64 slices, in-plane resolution = 2 × 2 mm, slice thickness = 2 mm, field of view = 256 × 256 mm, matrix size = 128 × 128 (voxel size = 2 × 2 × 2 mm^3^), repetition time = 9500 ms, echo time = 103 ms, b-value = 1000 s/mm^2^. Four images with no diffusion weighting were also acquired (b-value = 0 s/mm^2^). DTI images were then preprocessed and analysed according to a pipeline used previously in the group, in order to obtain FA and MD maps. Detailed information about DTI preprocessing (Smith, 2002; Behrens *et al.*, 2003; Smith *et al.*, 2004; Woolrich *et al.*, 2009) and data analysis pipeline (Nichols and Holmes, 2002; Smith *et al.*, 2004, 2006; Smith and Nichols, 2009), can be found in the Supplementary material. In general, after TBI, FA decreases and MD increases relative to control values (Kinnunen *et al.*, 2011).

We first compared FA and MD values between two groups: (i) controls; and (ii) acute TBI patients, with whole brain voxel-wise analysis.

Using our criteria to classify patients as having impaired or preserved balance (described above), we assessed a voxel-wise whole brain analysis, with a three-level between-group factor, i.e. (i) controls; (ii) impaired balance acute TBI; and (iii) preserved balance acute TBI.

Using our criteria to classify TBI patients as with/without vestibular agnosia (described above), we ran a voxel-wise whole brain analysis with a three-level between-groups factor: (i) controls; (ii) TBI patients with vestibular agnosia; and (iii) TBI patients without vestibular agnosia.

We also correlated vestibular-mediated posturography, vestibular-perceptual thresholds, and vestibular-ocular reflex thresholds with FA and MD values.

### Data availability

The authors confirm that the data supporting the findings of this study are available within the article and its Supplementary material. Raw data that support the findings of this study are available from the corresponding author, upon reasonable request.

## Results

We screened 918 patients via the medical notes, clinically assessed 146 and recruited 37 acute TBI patients. Table 1 shows patients’ demographics and clinical measures including the Mayo TBI severity classification (Malec *et al.*, 2007). Table 2 shows assessments of reflex vestibular function. We also tested 37 age matched healthy controls (mean ± SD: 40.8 ± 15 years, 21 females).

The 146 patients were composed of patients who potentially met our inclusion/exclusion criteria, as well as some patients who potentially did not meet our criteria but who we were asked to review by the therapy team for imbalance and/or dizziness. From this core group of patients, we then aimed to recruit only those patients who fulfilled our selection criteria. Of the 146 patients we assessed, the two commonest reasons for non-recruitment were: (i) unwillingness or inability to obtain consent or assent, including because of language barriers or no identifiable next of kin (22%); and (ii) medically unstable, obviating laboratory assessment (14%). Signs of an acute unilateral peripheral vestibular loss (see ‘Clinical assessment’ section) was found on bedside examination in 7% of assessed patients. A full list of the reasons for non-recruitment in examined cases is given in Supplementary Table 1.

We recruited 37 acute TBI patients in total for whom all behavioural and MRI data were acquired in the acute phase. We only used 30 patients in our MRI analysis since after the first seven MRI scans were performed, we began to use a different scanner for logistical reasons (scanner access) and hence, to avoid difficulties of comparing results between scanners, we only analysed MRI data for 30 patients. We recruited 37 matched controls who had behavioural and MRI scan data acquired except for one control who did not have an MRI. We were not able to perform this because of the COVID19 pandemic; hence, we analysed 36 control MRI scans.

### Laboratory testing

#### Vestibular threshold testing: vestibular agnosia in acute traumatic brain injury

**Figure 2:**
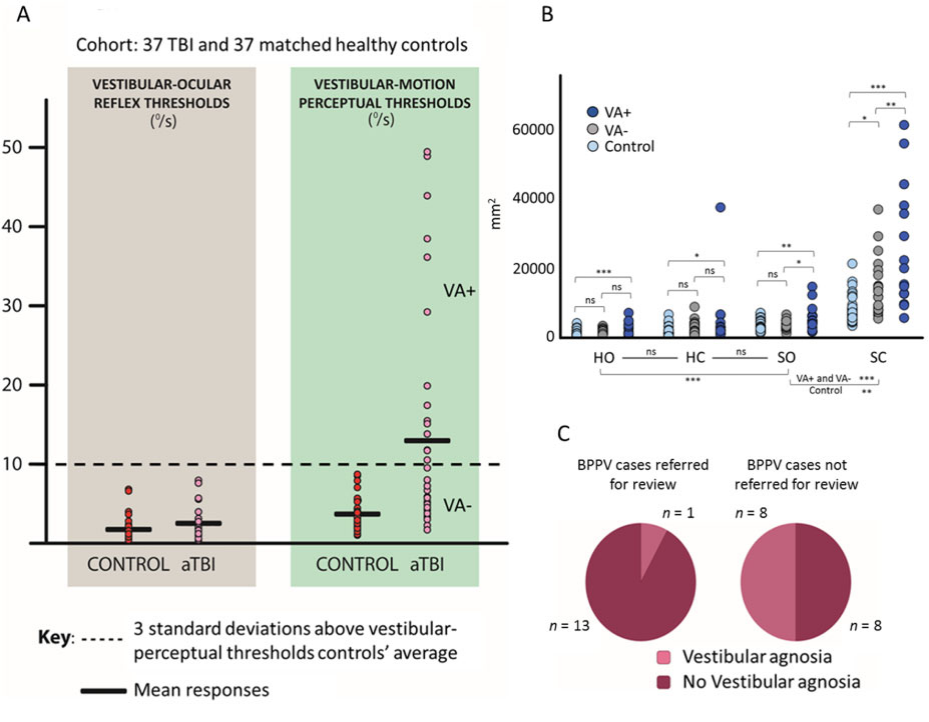
**Vestibular threshold testing.** (**A**) Vestibular agnosia in acute TBI. Vestibular-ocular (*left*) and vestibular-perceptual (*right*) thresholds to angular acceleration, in healthy controls (dark red) and acute TBI patients (light pink): the acceleration thresholds are displayed in terms of the equivalent instantaneous angular velocity at the time of the threshold detection. Vestibular-ocular thresholds (expressed in degrees per second), correspond to the minimum angular velocity needed to elicit a vestibular-ocular response (first slow-phase of a nystagmus with minimum of two slow and fast phase components). Perceptual thresholds (expressed in degrees per second), correspond to the minimum angular velocity needed to induce the perception of self-motion in the correct direction as assessed by the MOBS procedure. (**B**) Imbalance in acute TBI assessed via posturography. Sway expressed in square millimetres as the area of the 95% bivariate confidence ellipse of the total displacement of the centre of pressure, in the four posturography conditions (HO = hard surface with eyes open; HC = hard surface with eyes closed; SO = soft surface with eyes open; SC = soft surface with eyes closed), in controls (light blue), acute TBI patients without vestibular agnosia (VA-, grey), and acute TBI patients with vestibular agnosia (VA+, blue). ns = not significant. *P < 0.05; **P < 0.01; ***P < 0.001. (**C**) Clinically apparent vestibular agnosia masks the presence of BPPV in acute TBI. *Left*: Patients with BPPV, diagnosed after being referred by the ward clinical staff (*n = *14). *Right*: Patients with BPPV, who were not referred by the ward clinical staff, but diagnosed by our systematic screening on the trauma ward (*n = *16). The dark red sectors represent the proportions of patients who reported dizziness during manoeuvres, i.e. they did not have vestibular agnosia. The light pink sectors represent the patients with vestibular agnosia, i.e. they denied dizziness on direct questioning, during manoeuvres that triggered a vestibular nystagmus indicative of BPPV.

Figure 2A shows the elevated vestibular-perceptual thresholds in acute TBI. An ANOVA showed a significant main effect of: (i) threshold [*F*(1,68) = 30.25, *P < *0.001; η_p_^2^ = 0.31]; (ii) group [*F*(1,68) = 14.56, *P < *0.001; η_p_^2^ = 0.18]; and (iii) their interaction [*F*(1,68) = 13.40, *P < *0.001; η_p_^2^ = 0.16]. *Post hoc* comparisons showed that patients’ vestibular-perceptual thresholds (12.92 ± 14.14°/s) were higher (*P < *0.001) than the vestibular-ocular thresholds (2.52 ± 2.03°/s). Controls’ perceptual (3.87 ± 2.13°/s) and vestibular-ocular (1.78 ± 1.49°/s) thresholds were not different (*P = *0.19). Although vestibular-ocular thresholds were marginally elevated in the acute TBI group (*P = *0.08; Fig. 2A), acute TBI patients’ vestibular-perceptual thresholds were dramatically elevated compared to controls (*P < *0.001; Fig. 2A) (similar results were obtained in a pilot study whose data are reported in the Supplementary material). A perceptual threshold above 10.26°/s (3 SDs above the controls’ mean vestibular-perceptual threshold) indicated the patient was classified as having a vestibular agnosia, versus those with a value below 10.26°/s classified as not having vestibular agnosia. Using a conservative value of 3 SDs above the control group average (to ensure clear demarcation between impaired and non-impaired patients), 15 of 37 patients with acute TBI had a vestibular agnosia.

#### Reaction time testing, cognitive scores and vestibular agnosia

Patients were significantly slower than controls in the visual reaction time task, albeit by only 0.05 s [acute TBI 0.41 ± 0.08 s versus controls 0.36 ± 0.06 s; *F*(1,62) = 10.37, *P < *0.01; η_p_^2^ = 0.14], consistent with previous findings in TBI patients (Bonnelle *et al.*, 2011). Notably, reaction times were not different between TBI patients with and without vestibular agnosia [0.44 ± 0.09 s versus 0.40 ± 0.07 s; *t*(28) = 1.14, *P = *0.26]. Overall, these findings show that slower reaction times are insufficient to explain elevated vestibular-perceptual thresholds in TBI patients with vestibular agnosia.

Although we found modest albeit significant correlations between vestibular perceptual threshold values and three ACE-R subscale scores (attention *r* = −0.39, *P = *0.018; fluency *r* = −0.373, *P = *0.025; language *r* = −0.367, *P = *0.028), none survived correction for multiple comparisons.

#### Imbalance in acute traumatic brain injury assessed via posturography

Figure 2B shows that TBI patients with vestibular agnosia were more unstable than controls in all conditions, while TBI patients without vestibular agnosia were more unstable than controls only in the vestibular-mediated condition (soft surface with eyes closed). Moreover, TBI patients with vestibular agnosia were more unstable than those without vestibular agnosia on both soft surface conditions. Specifically, the ANOVA showed a significant effect of condition [*F*(3,213) = 137.43, *P < *0.001; η_p_^2^ = 0.66], of group [*F*(2,71) = 15.94, *P < *0.001; η_p_^2^ = 0.31] and of the interaction condition by group [*F*(6,213) = 16.04, *P < *0.001; η_p_^2^ = 0.31].

#### The link between vestibular symptoms and objective deficit in acute traumatic brain injury

Acute TBI patients with and without vestibular agnosia reported moderate dizziness symptoms, with DHI scores of 22.53 ± 17.05 for TBI patients with vestibular agnosia and 29.73 ± 22.49 for those without vestibular agnosia. Importantly, the DHI did not differ between TBI patients with and without vestibular agnosia [*t*(35) = −1.05, *P = *0.30], and DHI scores did not correlate with vestibular-perceptual thresholds in acute TBI (*r* = −0.16, *P = *0.33). Similarly, the ABC scale, did not discriminate between patients with objectively impaired balance (via posturography) versus those with preserved balance [ABC scores for impaired versus preserved balance: 77.19 ± 17.28 versus 72.91 ± 23.81; *t*(32) = 0.51, *P = *0.61]. The ABC score was not correlated with posturography performance in the vestibular-mediated condition (*r* = −0.05, *P = *0.80). These data confirm our earlier reports (Sargeant *et al.*, 2018; Marcus *et al.*, 2019) that symptoms and signs are typically uncoupled in patients with acute TBI. Thus acute TBI patients with signs of an active peripheral vestibular condition (e.g. BPPV) may have no vertigo, and conversely, those with overt imbalance on examination may not complain of imbalance.

#### The impact of vestibular agnosia upon clinical recognition of benign paroxysmal positional vertigo

Between 30 August 2017 and 15 May 2018, 67 patients were clinically assessed either for inclusion in the study or were clinically assessed following referral to the clinical vestibular team from the ward therapists. Of these 67 patients, 30 had BPPV (45%), (Fig. 2C). Of these BPPV cases, a third had an attenuated vertigo perception (i.e. a vestibular agnosia) during diagnostic or treatment manoeuvres (Supplementary Video 1). Of the 30 patients (of 67) with BPPV, 16 were diagnosed by our screening process and 14 were referred for dizziness or imbalance by the therapy team. Only 7% of BPPV cases referred to us by the therapists had a clinically apparent vestibular agnosia, whereas, of BPPV cases we diagnosed by screening, 50% had a clinically apparent vestibular agnosia (Fig. 2C). Thus, vestibular agnosia reduces the probability of BPPV being identified by clinical staff (χ^2^ = 6.53, *df* = 1, *P < *0.02).

#### Vestibular thresholds and posturography in patients with and without benign paroxysmal positional vertigo

Patients with BPPV compared to those without, did not show any significant differences (i.e. *P *>* *0.05; two-tailed *t*-tests) in vestibular perceptual thresholds [*t*(35) = 0.16, *P = *0.87], VOR thresholds [*t*(32) = −0.15, *P = *0.88] or postural sway [*t*(35) = −1.11, *P = *0.27], indicating that a BPPV diagnosis, which was treated prior to any testing, had no functional impact on reflex or perceptual vestibular measures nor postural stability.

### Neuroimaging

#### DTI comparison between all patients and controls

**Figure 3:**
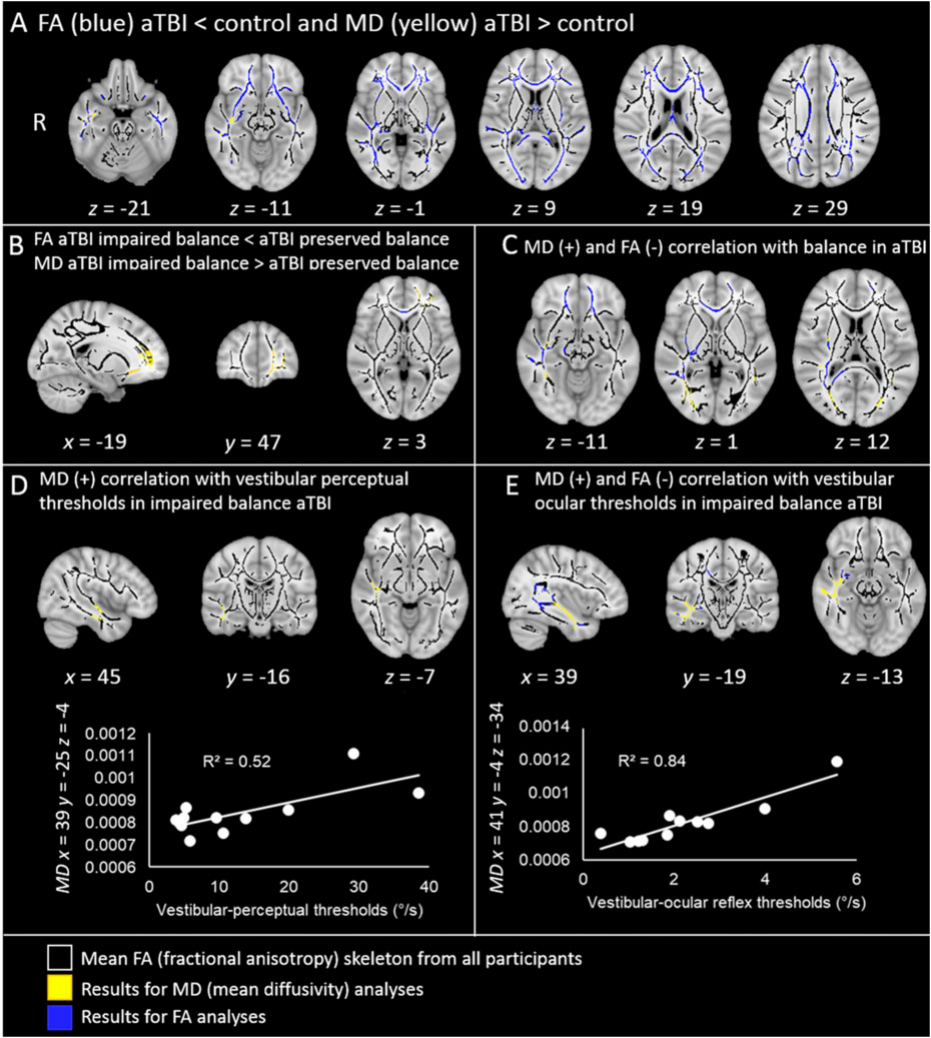
**Widespread white matter disruption following TBI and correlations with behavioural measures.** All contrasts are overlaid upon a standard MNI 152 T_1_ 1 mm brain atlas and the mean FA skeleton (black) with display thresholds set to range from 0.2 to 0.8. The results of FA tract-based spatial statistics contrasts (blue) and the results of MD tract-based spatial statistics contrasts (yellow), are thresholded at *P *<* *0.05, corrected for multiple comparisons. (**A**) Axial slices of the results of the FA contrast acute TBI < control (blue), and of the MD contrast between acute TBI > control groups (yellow). (**B**) Sagittal, coronal and axial slices of the results of the FA contrast between patients with impaired balance < patients with preserved balance (blue), and of the MD contrast between patients with impaired balance > patients with preserved balance (yellow). (**C**) Axial slices of the results of the contrast where MD values positively correlate with balance performance (yellow), and where FA values negatively correlate with balance performance in acute TBI patients (i.e. the higher the MD values, the more instability in acute TBI; the lower the FA values, the more instability in acute TBI). (**D**) *Top*: Orthogonal view of the areas in the inferior longitudinal fasciculus where patients with impaired balance (but not patients with preserved balance and controls) showed significant positive correlations between MD and vestibular-perceptual thresholds (i.e. the higher the MD value, the more severe the vestibular agnosia, in acute TBI with impaired balance). *Bottom*: The plot shows the positive correlation between MD and vestibular-perceptual thresholds (°/s) in the significant voxel in the inferior longitudinal fasciculus with the highest correlation with vestibular-perceptual thresholds (*x *=* *39, *y* = −25, *z* = −4). (**E**) *Top*: Orthogonal view of the areas where patients with impaired balance (but not patients with preserved balance and controls) showed significant correlations between vestibular-ocular reflex thresholds and MD (positive correlation, in yellow) and between vestibular-ocular thresholds and FA (negative correlations, in blue). *Bottom*: For illustrative purposes, the plot shows the positive correlation between MD and vestibular-ocular reflex thresholds (°/s), in the voxel with the highest correlation with vestibular-ocular reflex thresholds (*x *=* *41, *y* = −4, *z* = −34).

A voxel-wise whole brain comparison showed significantly lower FA in acute TBI patients (*n *=* *30) compared to controls (*n = *36) in a widespread bilateral network (detailed in Fig. 3A and Table 3). Areas of significantly higher MD were also found, albeit in a less extensive network.

**Table 3:** Contrasts

Contrasts differences between groups (*P* peak voxel)	Areas with significant *Z*-value threshold 0.05
FA aTBI < control^**^ (0.003)	CC (genu, body, splenium); fornix (column, body); retrolenticular internal capsule R, L; ant corona radiata R, L; sup corona radiata R, L; post corona radiata R, L; post thalamic radiation^a^ R, L; sagittal stratum^b^ R, L; external capsule R, L; cingulum (cingulate gyrus) R, L; fornix (cres) / stria terminalis R, L; sup long fasc R, L; uncinate fasc R, L; tapetum R
MD aTBI > control^*^ (0.04)	Ant corona radiata L; sagittal stratum^b^ R; external capsule R
**Contrasts differences between subgroups**	
FA VA+ < control^*^ (0.02)	CC (genu, body, splenium); ant limb internal capsule R; ant corona radiata R, L; sup corona radiata R, L; post corona radiata R; post thalamic radiation^a^ R; sagittal stratum^b^ R; external capsule R, L; fornix (cres) / stria terminalis R; sup long fasc R, L; sup front-occ fasc R; uncinate fasc R
MD VA+ > control^*^ (0.03)	Sagittal stratum^b^ R
FA VA- < control^*^ (0.02)	CC (genu, body, splenium); ant corona radiata R, L; sup corona radiata R, L; post corona radiata L; sagittal stratum^b^ R, L; external capsule R, L; cingulum (cingulate gyrus) L; sup long fasc R, L; uncinate fasc R, L
FA impaired balance aTBI < control^**^ (0.001)	CC (genu, body, splenium); fornix (column, body); cerebral peduncle R; ant limb internal capsule L; post limb internal capsule R; retrolenticular internal capsule R, L; ant corona radiata R, L; sup corona radiata R, L; post corona radiata R, L; post thalamic radiation^a^ R, L; sagittal stratum^b^ R, L; external capsule R, L; cingulum (cingulate gyrus) R; fornix (cres) / stria terminalis R, L; sup long fasc R, L; uncinate fasc R, L; tapetum R
MD impaired balance aTBI > control^**^ (0.008)	CC (genu, body, splenium); ant limb internal capsule R, L; retrolenticular internal capsule R, L; ant corona radiata R, L; sup corona radiata R, L; post corona radiata R, L; post thalamic radiation^a^ R, L; sagittal stratum^b^ R, L; external capsule R, L; fornix (cres) / stria terminalis R, L; sup long fasc R, L; sup front-occ fasc R; uncinate fasc R, L; tapetum R, L
FA impaired balance aTBI < preserved balance aTBI^*^ (0.04)	CC (genu); ant corona radiata L
MD impaired balance aTBI > preserved balance aTBI^*^ (0.03)	CC (genu); ant corona radiata L; external capsule L
**Whole brain correlation contrasts**	
Vestibular-mediated balance	
All participants FA to balance (−)^**^ (0.005)	CC (genu, body, splenium); fornix (column, body); cerebral peduncle R, L; ant limb internal capsule R; post limb internal capsule R, L; retrolenticular internal capsule R, L; ant corona radiata R, L; sup corona radiata R, L; post corona radiata R, L; post thalamic radiation^a^ R, L; sagittal stratum^b^ R, L; external capsule R, L; cingulum (cingulate gyrus) R; fornix (cres) / stria terminalis R, L; sup long fasc R, L; sup front-occ fasc R; uncinate fasc R; tapetum R, L
All participants MD to balance (+)^*^ (0.02)	CC (genu, body, splenium); ant limb internal capsule R, L; retrolenticular internal capsule R, L; ant corona radiata R, L; sup corona radiata R, L; post corona radiata R, L; post thalamic radiation^a^ R, L; sagittal stratum^b^ R, L; external capsule R, L; fornix (cres) / stria terminalis R, L; sup long fasc R, L; sup front-occ fasc R; uncinate fasc R; tapetum R, L
aTBI FA to balance (−)^*^ (0.04)	CC (genu, body, splenium); cerebral peduncle R; post limb internal capsule R; retrolenticular internal capsule R; ant corona radiata R, L; sup corona radiata R; post corona radiata R; post thalamic radiation^a^ R; sagittal stratum^b^ R; external capsule R; sup long fasc R; tapetum R
aTBI MD to balance (+)^*^ (0.04)	CC (splenium); retrolenticular internal capsule R; sup corona radiata R; post corona radiata R, L; post thalamic radiation^a^ R; sagittal stratum^b^ R; external capsule R; fornix (cres) / stria terminalis R; sup long fasc R, L
VA+ MD to balance (+) (0.063)	^#^Post thalamic radiation^a^ R; ^#^sagittal stratum^b^ R; ^#^external capsule R
Vestibular-perceptual thresholds	
All participants MD to VPT (+)^*^ (0.04)	Sagittal stratum^b^ R
Impaired balance aTBI FA to VPT (−) (0.07)	^$^Post thalamic radiation^a^ R; ^$^sagittal stratum^b^ R
Impaired balance aTBI MD to VPT (+)^*^ (0.05)	Sagittal stratum^b^ R
Vestibular-ocular reflex thresholds	
Impaired balance aTBI FA to VOR (−)^*^ (0.02)	CC (genu, body, splenium); ant limb internal capsule R; retrolenticular internal capsule R; ant corona radiata R; sup corona radiata R; post corona radiata R; post thalamic radiation^a^ R; sagittal stratum^b^ R; external capsule R; Fornix (cres) / stria terminalis R; sup long fasc R; sup front-occ fasc R; uncinate fasc R
Impaired balance aTBI MD to VOR (+)^**^ (0.007)	Retrolenticular internal capsule R; sagittal stratum^b^ R; external capsule R

−/+ = negative/positive correlation contrast between DTI parameter and behavioural measure; ant = anterior; CC = corpus callosum; fasc = fasciculus; front-occ = fronto-occipital; L = left; long = longitudinal; post = posterior; R = right; sup = superior; VA+/− = patients with/without vestibular agnosia; VPT = vestibular-perceptual thresholds.

a Post thalamic radiation, includes optic radiation.

b Sagittal stratum, includes inferior longitudinal fasciculus and inferior front-occipital fasciculus.

* *P *<* *0.05.

** *P *<* *0.01.

# Tendency, threshold set at 0.065.

$ Tendency, threshold set at 0.075.

#### Comparing white matter microstructure between groups according to balance performance

A voxel-wise whole brain analysis with a three-level between-group factor (controls, acute TBI impaired balance, acute TBI preserved balance), showed that acute TBI patients with impaired balance (*n = *11) displayed significantly lower FA and significantly higher MD compared to acute TBI patients with preserved vestibular-mediated balance (*n = *19) in a left anterior network comprising the genu of the corpus callosum, the left anterior corona radiata, and the left external capsule (Fig. 3B and Table 3). See Table 3 for other significant contrasts.

#### Correlating balance with DTI parameters

Vestibular-mediated balance performance (i.e. soft surface-eyes closed condition) was negatively correlated with FA values across all participants (acute TBI and controls together) in a widespread bilateral white matter network (Table 3). Similar findings were found for increased MD albeit in a less extensive network (Table 3).

These results were primarily driven by the acute TBI group, such that when running the correlations with group (acute TBI versus control) as a covariate, acute TBI patients showed significant correlations between DTI and vestibular-mediated sway performance (correlations were negative for FA and positive for MD) in numerous, albeit predominantly right hemisphere, white matter tracts (Fig. 3C and Table 3), whereas the control group alone showed no DTI-balance performance correlations for either MD or FA.

A voxel-wise whole brain correlation analysis with three-level between-group covariate (controls, imbalanced and preserved balance patients) showed no significant correlations between vestibular-mediated balance task and FA or MD in neither preserved or impaired balance patient group (Table 3).

When running a voxel-wise whole brain correlation analysis with three-level between-group covariate (controls, TBI patients with and without vestibular agnosia), a trend towards significance was found in TBI patients with vestibular agnosia when correlating DTI parameters (both MD and FA) with vestibular-mediated sway performance (Table 3; MD, *P*-value in the peak voxel = 0.06; FA *P*-value in the peak voxel = 0.09). No such correlation was found for the TBI without vestibular agnosia patient group. These findings (Table 3) suggest that damage in the right posterior thalamic radiation, right sagittal stratum, and right external capsule are implicated in imbalance in vestibular agnosia patients.

#### Correlating vestibular perception with DTI parameters and its link to imbalance

We correlated vestibular-perceptual thresholds in all patients with DTI parameters (FA and MD; Table 3). This showed that higher MD values (i.e. impaired white matter microstructure) in the right inferior longitudinal fasciculus correlated with higher vestibular perceptual thresholds (worse performance) across all participants. Importantly, however, this correlation was driven by patients with impaired vestibular mediated balance (sway in condition soft surface with eyes closed, Fig. 2B). In fact, when running the correlations with group as a covariate (controls, impaired and preserved balance patients), only impaired balance patients showed significant correlations between MD values in the right inferior longitudinal fasciculus and vestibular perceptual thresholds (Fig. 3D and Table 3). A trend towards significance in the same region was found when correlating vestibular perceptual thresholds and FA, but again only in impaired balance patients (negative correlation, FA *P*-value in the peak voxel = 0.07). There were no correlations between MD or FA and vestibular-perceptual thresholds in controls or patients with preserved balance. Finally, no significant correlations with vestibular parameters (perception or reflex) were found in the left inferior longitudinal fasciculus, confirming the right lateralized vestibular cortical representation (Dieterich *et al.*, 2003). This result suggests that, in acute TBI patients, vestibular-mediated postural instability, most prominent when standing in the dark and on an uneven (or soft) surface, is mediated by damage to brain areas that are also involved in mediating the vestibular perception of self-motion.

#### Correlating vestibular-ocular reflex thresholds with DTI parameters

In patients with impaired balance only, VOR thresholds correlated negatively with FA and positively with MD in a right white matter network, which included voxels in the right inferior longitudinal fasciculus (Fig. 3E and Table 3) that partially overlapped those that correlated with vestibular perceptual thresholds (and again, only in impaired balance patients). No other correlations were found between FA and MD values and vestibular-ocular reflex in controls or patients with preserved balance.

## Discussion

Our main findings were: (i) the balance impairment in acute TBI is primarily of a vestibular-dependent pattern and the degree of imbalance correlates with widespread bilateral and predominantly frontal white matter microstructural parameters on DTI; (ii) vestibular agnosia (the attenuation of vestibular sensation of self-motion, despite intact peripheral and reflex vestibular function) is frequent in acute TBI patients, at least in our cohort, where one-third of cases were affected; (iii) acute TBI patients with vestibular agnosia were more unbalanced than patients without vestibular agnosia; (iv) in those patients with impaired balance, DTI metrics of white matter microstructure in the right inferior longitudinal fasciculus correlated with the degree of vestibular agnosia; and (v) vestibular agnosia markedly reduces clinician awareness of the presence of active balance disorders given that acute TBI patients with BPPV were seven times less likely to be referred for treatment if there was concurrent vestibular agnosia.

### Imbalance in acute traumatic brain injury

Remarkably, this is the first prospective study to assess balance and vestibular function in patients with acute TBI. We confirm with laboratory analyses, our previous cross-sectional clinical-bedside studies’ findings (Sargeant *et al.*, 2018; Marcus *et al.*, 2019) that postural imbalance in acute TBI is both common (affecting over 80% of ambulant acute TBI patients) and displays a vestibular-dependent pattern. Our previous clinical report (Marcus *et al.*, 2019) showed that examination of acute TBI patients with intact peripheral vestibular functioning, follows a pattern reported by Brandt in acute peripheral vestibulopathy patients (Brandt *et al.*, 1999), i.e. patients were more stable walking than standing still. In our current acute TBI cohort, our data imply patients manifest a central vestibular ataxia.

Roughly half of acute TBI cases have BPPV, and since BPPV causes imbalance and falls (Oghalai *et al.*, 2000), we screened and treated BPPV in every case we saw, irrespective of their complaint, thus it is unlikely that BPPV contributed to our findings. Acute peripheral vestibular loss, affecting up to 19% of acute TBI cases in previous reports (Marcus *et al.*, 2019), was an exclusion for our study. Of the 146 patients examined for this study, 7% had unilateral peripheral vestibular loss, but notably none had evidence of a bilateral peripheral vestibular impairment, which likely relates to the survivability of an injury affecting both petrous temporal bones.

### The clinical syndrome of vestibular agnosia

We provide the first quantitative demonstration of markedly attenuated self-motion perception or ‘vestibular agnosia’ in patients with acute TBI. The term vestibular agnosia distinguishes this deficit of vestibular-motion perception from our previous report of a lateralized vestibular-spatial impairment in acute focal stroke affecting the right temporo-parietal junction (Kaski *et al.*, 2016). A qualitative obtundation of the vestibular sensation of self-motion has been reported previously in some elderly patients in whom small vessel disease is a common occurrence (Seemungal, 2005; Imbaud Genieys, 2007; Chiarovano *et al.*, 2016). Notably, the average age of our acute TBI cohort was 42 years, with the youngest case being 18 years old, eliminating premorbid confounds of age-related brain conditions such as neurodegeneration or small vessel disease.

Despite our observation that vestibular agnosia is common in acute TBI, this clinical syndrome has evaded detailed scientific scrutiny up until now, perhaps because of how such patients present and to whom. Thus, patients presenting with falls without vertigo are assessed by falls clinics that do not routinely assess vestibular diagnoses (Seemungal, 2016), whereas patients with vertigo—who are referred to vestibular clinics—by definition do not have vestibular agnosia. Hospitalized acute TBI patients are typically managed by surgical specialities who focus upon life-saving surgical interventions. Another argument is that vestibular agnosia can only be quantified by vestibular perceptual testing (Seemungal *et al.*, 2004), which is a research test, and currently there are no commercially available vestibular perceptual tests for routine clinical use.

Vestibular agnosia could simply be a specific manifestation of a general anosognosia, reported to affect 97% of acute TBI cases (Sherer *et al.*, 1998). Additional testing was not possible in our acute patient group because of the burden of testing. Anecdotally, at least one acute TBI patient with vestibular agnosia denied seeing the room move, reporting it to be stable despite an ongoing nystagmus due to BPPV, a finding previously noted also anecdotally (Seemungal, 2005). This suggests that at least in some cases of acute TBI with vestibular agnosia (which we assessed in the dark), there is a form of a global akinetopsia, typically reported in patients with bilateral extrastriate cortical lesions (Shipp *et al.*, 1994). None of our vestibular agnosia patients had bilateral contusions affecting the extrastriate cortex and indeed in some cases with severe vestibular agnosia there were few or even no contusions (although in these cases, significant diffuse axonal injury could have resulted in disconnected inter-hemispheric regions).

### The neuroimaging correlates of vestibular agnosia and its link with imbalance

Our neuroimaging findings did not support our hypothesis that vestibular agnosia was related to the disruption of a bihemispheric network, either because our hypothesis was incorrect, i.e. that vestibular agnosia is localizable to a specific brain region, or only partially incorrect, i.e. that damage to multiple brain areas and/or combinations of brain areas can result in the clinical syndrome of vestibular agnosia. In either case, our imaging null result for this specific question could relate to an insufficient sample size, particularly given our conservative approach to use whole brain analyses and not a region of interest approach. Despite our current result, the notion of a distributed network mediating the vestibular sensation of self-motion is supported by several previous findings. First, Nigmatullina *et al.* (2015) showed a correlation between a vestibular-motion perceptual parameter and an extensive white matter network in healthy individuals. Second, previous tractography studies in healthy participants (Kirsch *et al.*, 2016; Wirth *et al.*, 2018) have shown a bihemispheric pattern of connectivity between putative vestibular cortical areas including the parieto-insular vestibular cortex (PIVC) and the posterior insular cortex (PIC). Indeed, these bihemispheric vestibular cortical regions were connected via the corpus callosum (Kirsch *et al.*, 2016; Wirth *et al.*, 2018), a region commonly affected in TBI (Ham and Sharp, 2012). Although we found extensive callosal damage in our acute TBI patients compared to controls, we were not able to discern a different pattern of callosal damage between acute TBI patients with and without vestibular agnosia.

### Vestibular agnosia and imbalance are mechanistically linked

Concerning the link between vestibular agnosia and imbalance in acute TBI, we considered three possibilities. First, that the two are independent; second, they are linked but non-mechanistically; and third, that vestibular agnosia is mechanistically linked, at least in part, to imbalance. Our data suggest that vestibular agnosia is mechanistically linked to imbalance in acute TBI and this link is mediated by damage to the right inferior longitudinal fasciculus. Specifically, we found that only in acute TBI patients with impaired balance that DTI parameters (FA and MD) in the right inferior longitudinal fasciculus correlated with vestibular perceptual thresholds. No such relationship was found for patients with preserved balance or healthy controls.

Perhaps significantly, in the most extensive survey of human vestibular sensations in response to electrocortical stimulation, Kahane *et al.* (2003) showed that right inferior longitudinal fasciculus stimulation provoked a vestibular sensation of yaw-plane head spinning. This elegant study (in which vestibular sensations were rigorously described) also found such yaw-plane sensations were often provoked by electrical stimulation to loci in the superior and middle temporal gyri that are juxtaposed and connected by the inferior longitudinal fasciculus. Interestingly the inferior longitudinal fasciculus is prominently affected in patients with progressive supranuclear gaze palsy (PSP), a neurodegenerative extrapyramidal disorder with early-onset postural instability (Whitwell *et al.*, 2011; Agosta *et al.*, 2014). Additionally, the pattern of posturography impairment in PSP mirrors that seen in acute TBI patients, i.e. it is primarily of a vestibular-dependent posturography pattern (Ondo *et al.*, 2000; Cronin *et al.*, 2017).

Perhaps more surprising was our finding of a link between VOR thresholds and DTI parameters, again only in unbalanced patients, and again in the inferior longitudinal fasciculus in a discrete albeit partially overlapping region to that linked to vestibular perceptual thresholds. Previous lesion studies have however shown top-down modulation of the VOR, albeit of modest effect size, with primarily right hemisphere lesions in the temporal and parietal lobes in humans (Ventre-Dominey *et al.*, 2003; Ventre-Dominey, 2014). That disruption of the right inferior longitudinal fasciculus correlated with both vestibular perceptual and vestibular ocular thresholds and only in those acute TBI patients with impaired vestibular-mediated balance, suggests a convergence of multiple vestibular related functions—both sensory (vertigo sensation) and motor (balance control and VOR)—in the right hemisphere. The notion that this region mediates a vestibular sensorimotor convergence is supported by a previous study showing that DTI metrics in the inferior longitudinal fasciculus predicted the age-related decline in performance of a visuomotor task (Voineskos *et al.*, 2012), indicating the involvement of this fasciculus in sensorimotor integration in general.

Although speculative, our finding of a convergence of vestibular sensory and motor control systems at cortical level may be advantageous from an ecological perspective. For example, brain areas mediating self-motion sensation that also mediate top-down control of the VOR may be useful during pathological stimulation of the inner ear mechanism where vertigo sensation would be aggravated by the provoked nystagmus (via induced oscillopsia). Hence mechanisms that suppress vertigo sensation both in the dark (VOR-independent) and light (VOR-dependent) would be ideally placed to facilitate recovery from peripheral vestibular lesions in a process called vestibular compensation.

### Limitations

We recruited only acute TBI cases with clinically preserved peripheral and brainstem reflex vestibular functioning, thus our data are not applicable to those acute TBI patients with impaired peripheral functioning (Marcus *et al.*, 2019). Our study did not recruit the most severe surviving patients since we required patients to be well enough to at least provide assent and perform simple tests. It is likely though that our findings of a vestibular agnosia will be even more common with a greater severity of brain injury, potentially important when providing long term rehabilitation for these cases since cryptogenic BPPV sans vertigo may provoke falls or episodic nausea (see next section).

### The clinical impact of vestibular agnosia

Our data show that vestibular agnosia affects clinical care of acute TBI patients. Specifically, vestibular agnosia reduced pick up rates of a common inner ear problem (BPPV) 7-fold, due to the lack of patients’ vertigo complaint. Our current data confirm our previous cross-sectional report (Sargeant *et al.*, 2018) that in acute TBI, subjective patient reports are unreliable as a surrogate measure for vestibular dysfunction in acute TBI. Objective measures of vestibular function, including reflex, perceptual and balance assessment, are necessary to accurately describe the vestibular deficit. This approach is at variance with the traditional clinical teaching that defining a patient’s symptoms are key to the underlying diagnosis. That the traditional symptom-based approach does not work in acute TBI is evidenced by our finding that the majority of BPPV diagnoses were missed by the major trauma team in acute TBI cases with concurrent vestibular agnosia.

Interestingly, vestibular agnosia patients are not immune from nausea. The index case (a 33-year-old male) that first alerted the senior author to the presence of vestibular agnosia in acute TBI was a patient who was referred solely for imbalance and positional nausea, and in whom a positional manoeuvre revealed BPPV without vertigo. Although treating BPPV is likely beneficial in acute TBI, the optimal time to treat is unknown since one retrospective case series showed a 67% recurrence rate versus only 14% for non-traumatic BPPV (Gordon *et al.*, 2004). An on-going feasibility study, however, will help to answer the question of when to treat BPPV following acute TBI (http://www.isrctn.com/ISRCTN91943864).

### Additional implications and conclusion

An immediate clinical implication is that patients at risk of vestibular agnosia (e.g. TBI, dementia, diffuse small vessel disease) who have concurrent inner ear disturbances, will not report vertigo, increasing the chance of treatable inner ear diagnoses going undetected, and, more pertinently, increasing the risk of falls. Vestibular agnosia may contribute to the observation that 10% of unbalanced elderly patients in the community have undiagnosed BPPV as these patients do not complain of vertigo (von Brevern *et al.*, 2007). Indeed, the term BPPV may bias clinicians away from making its diagnosis in patients without vertigo, lending support to its replacement with other terms [e.g. peripheral vestibular positional nystagmus (PVPN)]. An appreciation of vestibular agnosia should thus spur clinical studies to assess the impact of acute and prospective screening for common vestibular conditions in patients at risk of vestibular agnosia, such as TBI, elderly fallers and dementia. Such studies may help to refine current national guidelines for elderly fallers (Seemungal, 2016) that currently do not mention BPPV, a potentially injurious omission, since BPPV, with or without provoked vertigo, causes falls (Oghalai *et al.*, 2000) and treating BPPV reduces falls (Ganança *et al.*, 2010).

Finally, vestibular agnosia is important for retrospective assessment of TBI cases, whether for research studies or for medico-legal reports, the latter a common and important consideration in acute TBI cases. As noted, significant vestibular dysfunction may occur without vertigo in TBI patients, but also, patients may present with a delayed onset of vertigo symptoms post-TBI, providing a clinical conundrum when assessed retrospectively. Importantly, a vestibular condition caused by head trauma may only begin to trigger vertigo once the vestibular agnosia recovers. Our ongoing prospective follow-up of our acute TBI cohort will help to answer this issue. That this study is, to our knowledge, the only acute prospective study assessing vestibular dysfunction in TBI, indicates that much of the clinical literature assessing vestibular dysfunction in TBI will be biased by retrospective assessment, indicating the need for new prospective acute study data to replace previous retrospective reports.

To conclude, we provide the first quantitative demonstration of a vestibular cognitive deficit in acute TBI patients termed vestibular agnosia and link this clinical finding with impaired balance due to the disruption of white matter microstructure in the inferior longitudinal fasciculus in the right temporal lobe. Vestibular agnosia is distinct from a previously described vestibular-spatial deficit that is mediated by the right temporoparietal junction (Kaski *et al.*, 2016). The natural history of vestibular agnosia in TBI is currently unknown, however prospective studies are required to examine whether systematic screening for cryptogenic vestibular diagnoses in acute TBI, symptomatically masked by vestibular agnosia, changes clinical outcome.

## Supplementary Material

awaa386_Supplementary_Data

## Abbreviations

ABC: Activity-Specific Balance Confidence Scale
BPPV: benign paroxysmal positional vertigo
DTI: diffusion tensor imaging
FA: fractional anisotropy
MD: mean diffusivity
TBI: traumatic brain injury
VOR: vestibular ocular reflex
