# Supplementary material for: Vestibular agnosia in traumatic brain injury and its link to imbalance
Source: Brain. 2020 Dec 26;144(1):128–43. doi: 10.1093/brain/awaa386 (PMC7880674; doi:10.1093/brain/awaa386)
Supplement: awaa386_Supplementary_Data [file awaa386_supplementary_data.zip › brain-2020-01273-File008.pdf]

Supplementary material: 'Vestibular Agnosia in Traumatic Brain Injury and its Link to Imbalance'

Calzolari E, Chepisheva M, Smith RM, Mahmud M, Hellyer PJ, Tahtis V, Arshad Q, Jolly A, Wilson M, Rust H, Sharp DJ, Seemungal BM

Online-only material content:

1) **Supplementary Clinical video: supporting clinical video for Vestibular Agnosia**
2) **Supplementary methods: Vestibular perceptual and vestibular ocular reflex thresholds tests**
3) **Supplementary methods: visual reaction time task**
4) **Supplementary methods: posturography**
5) **Supplementary methods: Dizziness Handicap Inventory and Activity-Specific Balance Confidence Scale**
6) **Supporting material – CT scans**
7) **Supplementary methods: Diffusion tensor imaging pre-processing and data analysis**
8) **Supplementary Table 1**
9) **Pilot data methods, Supplementary Figure 1**
10) **Results for pilot data, Supplementary Figure 2**

1) Clinical video: Supporting clinical video for Vestibular Agnosia uploaded with manuscript

The clinical video demonstrates how vestibular agnosia may mask subjective reports of vertigo that arise from common vestibular diagnoses, such as benign positional paroxysmal vertigo. That is, despite an intense nystagmus indicating labyrinthine stimulation (from the gravity-dependent movement of particles within the endolymph in the semi-circular canal), the patient reports no sensation of vertigo. Thus, patients with vestibular agnosia may have a treatable inner ear diagnosis which may go undetected by the medical team as the patient does not complain of vertigo. Critically, this increases the patient's risk of falls. Vegetative symptoms of nausea and vomiting may sometimes be the only indication of a peripheral vestibular disturbance in these patients.

2) Supplementary methods: Vestibular perceptual and vestibular ocular reflex thresholds tests

(i) Constant acceleration and MOBS staircase procedure.

Within each trial, participants were exposed to angular rotation in the yaw plane, at a specific constant acceleration (°/s^2), determined by a MOBS algorithm (Tyrrell and Owens, 1988), either to the right or to the left, from a stationary start, lasting a maximum of 5 seconds and followed by a maximum of 5 second deceleration (i.e. triangular velocity profile), and were asked to indicate, by button press, the direction of motion. If participants pressed a button before the end of the 5 second acceleration, the chair instantly started decelerating. Correct responses were defined as those identifying the correct direction within the 5 second acceleration period. Two independent MOBS staircase algorithms, one for left and one for right rotations, were used to determine the acceleration value for each trial and to calculate the

vestibular perceptual thresholds for the two directions. The order of leftward and rightward rotations was randomised. In each MOBS staircase procedure, the threshold was determined following the fulfilment of two termination criteria:

1) three response reversals (i.e. a change from correct detection of motion direction to error on the subsequent rotation, or vice versa);
2) the difference between the angular acceleration of the current rotation and the angular acceleration of the previous rotation at the final reversal, is less than 5% of the test range (lower boundary angular acceleration: 0.1°/s^2, higher boundary angular acceleration: 11.1°/s^2), otherwise a further two reversals are required.

Upon meeting both termination criteria for each MOBS staircase, the midpoint between the acceleration values of the last two rotations (one supra-threshold and one sub-threshold) was taken as vestibular perceptual threshold. This was obtained separately for left and right rotations. The average of left and right thresholds was taken as a measure of the mean perceptual threshold. For convenience, we converted vestibular-perceptual thresholds from angular acceleration (°/s^2) to angular velocity (°/s) to allow for better comparison with the vestibular-ocular reflex thresholds, obtained with the method below.

(ii) Increasing acceleration (steps) and average angular velocity.

In patients with a significant vestibular agnosia, perceptual thresholds were many times higher than VOR thresholds thus, in such cases, it was not possible to obtain VOR thresholds using the MOBS approach since the acceleration stimuli progressively increased in magnitude, away from the putative VOR threshold level, with each non-perceived stimulus. We therefore used a second method to ensure the measurement of VOR thresholds in which participants were exposed to angular rotations of increasing acceleration (0.3°/s^2 every 3 s) from a stationary start, either to the right or to the left. They were asked to press the corresponding button (right

or left) as soon as they perceived the movement and its direction. The chair continued to accelerate until a correct button response was made, or if 33 s (of which 30 s of acceleration and 3 s of rotation at constant maximum speed) had elapsed without a correct button press, or no button press. After a correct response or if no response was made during the test period over 33 seconds, the chair underwent a controlled deceleration to a stop over three seconds. Six rotations were performed in total, three right and three leftwards, in a randomised order. Simultaneously, we recorded eye movements using DC-coupled horizontal electrooculography acquired at a sampling rate of 250 Hz and used to obtain the vestibular-ocular threshold. We calculated the mean angular velocity (°/s) achieved at the time of the button press (a secondary measure of vestibular-perceptual threshold), and similarly the angular velocity achieved at the time of onset of the vestibular-nystagmus (vestibular-ocular threshold).

3) Supplementary methods: visual reaction time task

During each trial, an arrow – pointing left or right – appeared centrally on an LCD computer screen, 40 cm diagonal size, 1200 x 750 pixels resolution and 40 cm away. Participants pressed either a left or right button as quickly as possible indicating the arrow direction. The arrow stimulus extinguished upon button press. Interstimulus interval varied randomly in a range 2000 – 3000 ms. Equal right and left arrow trials (total 22 trials) were administered in random order.

4) Supplementary methods: posturography

We measured postural sway using a force platform (51.5 × 51.5 × 18 cm), containing four force sensors (Hottinger Baldwin Messtechnik – type U1Y – 100 kg – 2 mV/V), in a rectangular configuration to give a combined centre of pressure output (250 Hz sampling rate) for

mediolateral and anteroposterior directions. In particular, displacements of the net force on the platform were obtained through differential amplification of opposing pairs of transducers. Calibration was obtained using a 1.44 kg weight on four locations, at 394 mm distance from one edge of the platform to the other, in both anteroposterior and mediolateral directions. Participants' weight and height was recorded.

5) Supplementary methods: Dizziness Handicap Inventory and Activity-Specific Balance Confidence Scale

The DHI consists of 25 items evaluating the self-perceived impairment imposed by dizziness (Jacobson and Newman, 1990). The dizziness symptom score ranges from 0 (no symptoms) to 100 (maximum perceived disability). The ABC consists of 16 items measuring balance confidence in performing different activities without losing balance or experiencing a sense of unsteadiness (Powell and Myers, 1995). The confidence balance score ranges from 0 (no confidence of not losing balance – maximum perceived disability) to 100 (complete confidence of not losing balance – no perceived disability).

6) Supplementary methods: Diffusion tensor imaging pre-processing and data analysis

Diffusion tensor imaging pre-processing

First, we used linear transformations to register DTI images to the b = 0 image, in order to correct DTI data for head motion and eddy current distortions. Then a brain mask was generated by brain extracting the b = 0 image using FSL Brain Extraction Tool (Smith, 2002) from the FMRIB Software Library image processing toolbox (Smith *et al.*, 2004; Woolrich *et al.*, 2009).

We then used FMRIB's Diffusion Toolbox in FSL to fit a tensor model to the data. Finally, fractional anisotropy (FA) and mean diffusivity (MD) maps were generated voxelwise by applying this tensor model using the Diffusion Toolbox (Behrens *et al.*, 2003), for each participant.

Diffusion tensor imaging data analysis

Fractional anisotropy and mean diffusivity data were analysed voxelwise using Tract-based spatial statistics in the FMRIB Software Library (Smith *et al.*, 2004, 2006). First, all participants' FA and MD images were non-linearly aligned to a common 1mm FMRIB58 FA and MD standard space image. All aligned FA and MD images were then affine registered into the standard Montreal Neurological Institute (MNI) MNI152 1mm space. Two 4D images, one for FA and one for MD, were created by merging the aligned FA and MD images from all participants; mean fractional anisotropy and mean diffusivity 'skeletons' were also created by thinning the mean FA, representing the centre of all white matter tracts, and then projecting MD images in the FA skeleton. Finally, in order to exclude voxels with very low FA and those with high inter- inter-individual variability, the skeleton was thresholded at FA value of 0.2. Non-parametric permutation-based statistics were employed using randomisation with threshold-free cluster enhancement and 10000 permutations (Nichols and Holmes, 2002; Smith and Nichols, 2009). Results were corrected for multiple comparisons applying a threshold of $p \leq 0.05$. Age and gender were included as covariates.

7) **Supporting material – CT scans (340MB)**

https://1drv.ms/u/s!ArTNCM6FBLx8hIpdtI_gyluviZ8wKA?e=UMbwP8

8) **Supplementary Table 1**

Supplementary Table 1. Screening of patients and exclusion reason.

Exclusion reason	Number patients	%
Unwilling or unable to obtain consent (e.g. Language barrier) or assent (e.g. no identifiable next of kin)	24	22%
Medically unstable (e.g. severe sepsis or planned additional surgical interventions)	15	14%
Age > 65	11	10%
Out of area (e.g. foreign abode)	11	10%
Recreational drug or alcohol abuse/dependency	10	9%
Premorbid neurological diagnosis (e.g. stroke, dementia)	8	7%
Unilateral peripheral vestibular loss identified on examination	8	7%
Active psychiatric diagnosis (e.g. depression) &/or patients with agitation requiring sedation	8	7%
Chronic medical condition requiring active treatment (e.g. kidney disease)	7	6%
Orthopaedic &/or vascular injury affecting ability to stand	6	6%
Functional neurological syndrome (anxiety & functional gait)	1	1%
TOTALS	109	100%

9) Pilot data methods

Vestibular Threshold methods used in pilot data

Participants were exposed to angular rotations of increasing acceleration (0.5°/s^2 every 3 s) from a stationary start, either to the right or to the left. They were asked to press the corresponding button (right or left) as soon as they perceived the movement and its direction. The chair continued to accelerate until a correct button response was made, or if 30s has elapsed without a correct button press, or no button press. As no response was made during the test period over 30 seconds, the chair underwent a controlled deceleration to a stop over two seconds. Six rotations were performed in total, 3 right and 3 leftwards, in a randomised order. We calculated the mean angular velocity achieved at the time of the button press (vestibular-perceptual threshold), and similarly the angular velocity achieved at the time of onset of the vestibular-nystagmus (vestibular-ocular threshold).

After each rotation, the lights were turned on to ‘dump’ any residual low-level vestibular signal. White noise was delivered through earphones to mask auditory cues (Supplementary Fig. 1A).

Simultaneously, we recorded eye movements using DC-coupled horizontal electrooculography (ENG – Supplementary Fig. 1B) with ENG signals recorded at a sampling rate of 250 Hz to obtain the VOR threshold.

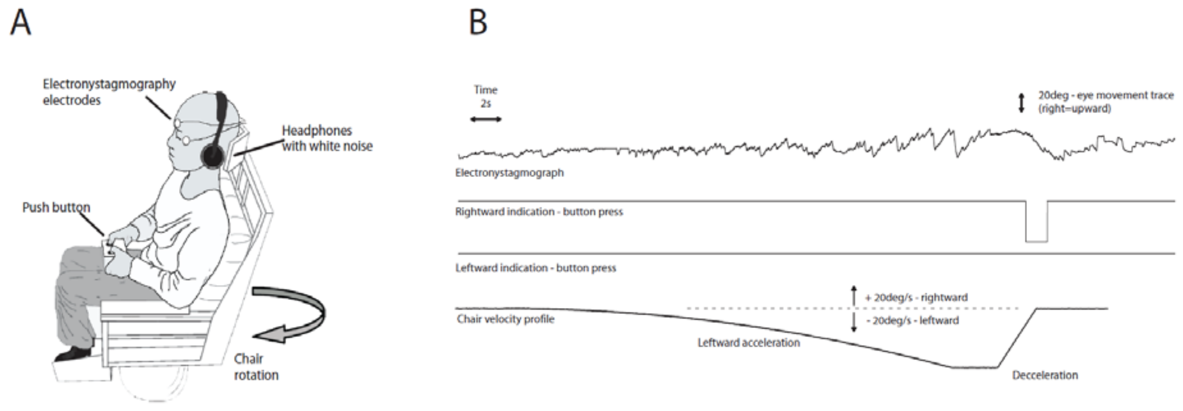

Supplementary Figure 1: Method for obtaining vestibular thresholds. A) Apparatus. Participants sat on a computer-controlled rotating chair (earth-vertical axis). Horizontal eye movements were recorded with electro-nystagmography (ENG). Subjects indicated their perceived direction of motion by pressing a button to indicate leftward or rightward motion. White noise was delivered through earphones. B) Raw traces for one rotation for Patient 9 (see also Patient 9's clinical video). The top trace shows the ENG signal. The second and third traces show the subject's button press to indicate perceived motion direction, right and left. In this example, the chair rotated from rest to the left, with incrementally increasing accelerations. The chair continues to accelerate until a correct button response is made or if 30s has elapsed without a correct button press, or no button press, as here. As no response was made during the test period over 30 seconds, the chair underwent a controlled deceleration to a stop over two seconds. This strong deceleration was quickly detected from the patient, who showed a clear stopping response (which is in the opposite direction of the previous rotation – which even for a normal subject is a right button press).

Statistical analysis

A repeated-measures ANOVA was performed to test differences between TBI patients with imbalance and healthy controls in the two thresholds measures (vestibular-ocular and vestibular-perceptual). The alpha-level was set at 0.05. Differences in the means for the significant interaction were then explored with two-tailed, paired-samples t-tests, Bonferroni corrected. To quantify the magnitude of the effects we report, we provide partial eta squared (η_p^2) values for F-tests, and d_z for paired t-tests.

10) Results for pilot data

We assessed both vestibular-ocular and vestibular-perceptual thresholds in 10 (mean age 37.8 years, SD 16.5 years, range 19-65 years, 3 females) patients with moderate-to-severe acute traumatic brain injury and 10 matched controls (mean age 38.0 years, SD 16.1, range 18-65 years, 5 females). As shown in Supplementary Fig. 2, TBI patients showed a markedly elevated vestibular-perceptual threshold compared to controls. This was confirmed with the ANOVA which revealed a significant main effect of threshold ($F_{1, 17} = 53.78$, $p < 0.00001$; $\eta_p^2 = 0.76$), of group ($F_{1, 17} = 37.97$, $p < 0.0001$; $\eta_p^2 = 0.69$), and of the interaction threshold by group ($F_{1, 17} = 37.76$ $p < 0.0001$; $\eta_p^2 = 0.69$). Paired-samples t-tests in the TBI group showed that ($t_8 = 6.67$, $p = 0.0002$; $d_z = 2.22$) the vestibular-perceptual thresholds (mean±SD = 48.09±20.03°s^{-1}) were higher than the vestibular-ocular thresholds (6.06±1.39°s^{-1}), while perceptual and VOR thresholds were not different in the control group ($t_9 = 2.13$, $p = 0.062$; vestibular-ocular = 3.43±2.58°s^{-1}; vestibular-perceptual = 7.14±5.72°s^{-1}). Moreover, whilst vestibular-ocular thresholds were equal in the two groups ($t_8 = 2.25$, $p = 0.055$ [α corrected with Bonferroni correction $\alpha = 0.0125$]), TBI patients showed dramatically increased vestibular-perceptual thresholds compared to healthy controls ($t_9 = 6.29$, $p = 0.0001$; $d_z = 1.99$).

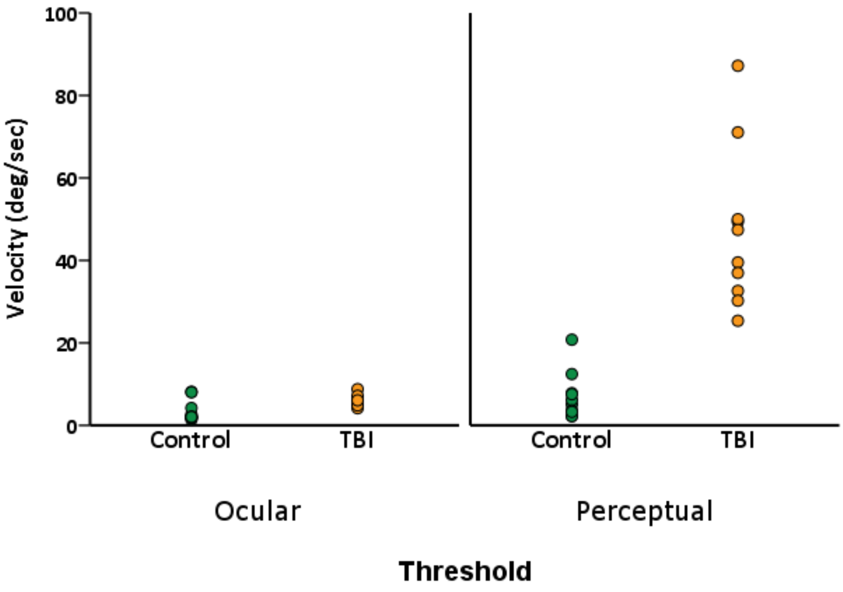

Supplementary Figure 2: Vestibular-ocular (left panel) and vestibular-perceptual (right panel) thresholds to angular acceleration, in healthy controls (green) and TBI patients (orange): The acceleration thresholds are displayed in terms of the equivalent instantaneous angular velocity at the time of the threshold detection. Vestibular-ocular thresholds (expressed in $^\circ s^{-1}$), correspond to the minimum angular velocity needed to elicit a vestibular-ocular response (first slow-phase of a nystagmus with minimum of two slow and fast phase components). Perceptual thresholds (expressed in $^\circ s^{-1}$), correspond to the minimum angular velocity needed to induce the perception of self-motion in the correct direction as recorded by the button press.

Stopping responses

Since the perceptual responses required a button press, a slowing in reaction time would result in an apparent (i.e. erroneous) elevated vestibular perceptual threshold. We found however that all subjects indicated their deceleration phase (perceived as a reversal of motion direction) within 1 second of stopping, implying that a delay in responding was not responsible for the observed impairment in vestibular threshold perception. In explanation, consider the kinematics of a stopping response. In a patient who does not detect the test acceleration, the

chair rotates up to maximum angular velocity of 100$^\circ s^{-1}$ (or 5.5$^\circ s^{-2}$) and then undergoes a controlled deceleration over 2s to a stop (i.e. an acceleration of 50$^\circ s^{-2}$). Hence the total change in acceleration is circa 55$^\circ s^{-2}$. That controls' average acceleration threshold was 1.1$^\circ s^{-2}$ (S.D. 0.45) indicates that a 'termination' stopping response acceleration is > 100 standard deviations above the normal acceleration threshold.
