# Supplementary material for: Vestibular agnosia in traumatic brain injury and its link to imbalance
Source: Brain. 2020 Dec 26;144(1):128–43. doi: 10.1093/brain/awaa386 (PMC7880674; doi:10.1093/brain/awaa386)
Supplement: awaa386_Supplementary_Data [file awaa386_supplementary_data.zip › brain-2020-01273-File009.pdf]

Supplementary Table 1. Screening of patients and exclusion reason.

Exclusion reason	Number of patients	% of patients
Unwilling or unable to obtain consent (e.g. Language barrier) or assent (e.g. no identifiable next of kin)	24	22%
Medically unstable (e.g. severe sepsis or planned additional surgical interventions)	15	14%
Age > 65	11	10%
Out of area (e.g. foreign abode)	11	10%
Recreational drug or alcohol abuse/dependency	10	9%
Premorbid neurological diagnosis (e.g. stroke, dementia)	8	7%
Unilateral peripheral vestibular loss identified on examination	8	7%
Active psychiatric diagnosis (e.g. depression) &/or patients with agitation requiring sedation	8	7%
Chronic medical condition requiring active treatment (e.g. kidney disease)	7	6%
Orthopaedic &/or vascular injury affecting ability to stand	6	6%
Functional neurological syndrome (anxiety & functional gait)	1	1%
TOTALS	109	100%
