# Supplementary material for: Vestibular agnosia in traumatic brain injury and its link to imbalance
Source: Brain. 2020 Dec 26;144(1):128–43. doi: 10.1093/brain/awaa386 (PMC7880674; doi:10.1093/brain/awaa386)
Supplement: awaa386_Supplementary_Data [file awaa386_supplementary_data.zip › brain-2020-01273-File012.pdf]

# Table 3. Contrasts

| **A) Contrasts differences between groups** | **P-value peak voxel** | **Areas with significant Z value threshold 0.05** |
|---|---|---|
| aTBI FA < control FA | **0.003**** | CC (genu, body, splenium)<br>Fornix (column and body of fornix)<br>Retrolenticular part of internal capsule R and L<br>Anterior corona radiata R and L<br>Superior corona radiata R and L<br>Posterior corona radiata R and L<br>Posterior thalamic radiation[a] R and L<br>Sagittal stratum[b] R and L<br>External capsule R and L<br>Cingulum (cingulate gyrus) R and L<br>Fornix (cres) / Stria terminalis R and L<br>Superior longitudinal fasciculus R and L<br>Uncinate fasciculus R and L<br>Tapetum R |
| aTBI MD > control MD | **0.04*** | Anterior corona radiata L<br>Sagittal stratum[b] R<br>External capsule R |
| **B) Contrasts differences between sub-groups** | | |
| VA+ FA < control FA | **0.02*** | CC (genu, body, splenium)<br>Anterior limb of internal capsule R<br>Anterior corona radiata R and L<br>Superior corona radiata R and L<br>Posterior corona radiata R<br>Posterior thalamic radiation[a] R<br>Sagittal stratum[b] R<br>External capsule R and L<br>Fornix (cres) / Stria terminalis R<br>Superior longitudinal fasciculus R and L<br>Superior fronto-occipital fasciculus R<br>Uncinate fasciculus R |
| VA+ MD > control MD | **0.03*** | Sagittal stratum[b] R |
| VA- FA < control FA | **0.02*** | CC (genu, body, splenium)<br>Anterior corona radiata R and L<br>Superior corona radiata R and L<br>Posterior corona radiata L<br>Sagittal stratum[b] R and L<br>External capsule R and L<br>Cingulum (cingulate gyrus) L<br>Superior longitudinal fasciculus R and L<br>Uncinate fasciculus R and L |
| VA- MD > control MD | 0.16 | |
| VA+ FA < VA- FA | 0.17 | |
| VA+ MD > VA- MD | 0.11 | |
| impaired balance aTBI FA < control FA | **0.001**** | CC (genu, body, splenium)<br>Fornix (column and body of fornix)<br>Cerebral peduncle R<br>Anterior limb of internal capsule L<br>Posterior limb of internal capsule R<br>Retrolenticular part of internal capsule R and L<br>Anterior corona radiata R and L<br>Superior corona radiata R and L<br>Posterior corona radiata R and L<br>Posterior thalamic radiation[a] R and L<br>Sagittal stratum[b] R and L<br>External capsule R and L<br>Cingulum (cingulate gyrus) R<br>Fornix (cres) / Stria terminalis R and L<br>Superior longitudinal fasciculus R and L<br>Uncinate fasciculus R and L<br>Tapetum R |
| impaired balance aTBI MD > control MD | **0.008**** | CC (genu, body, splenium)<br>Anterior limb of internal capsule R and L<br>Retrolenticular part of internal capsule R and L<br>Anterior corona radiata R and L<br>Superior corona radiata R and L<br>Posterior corona radiata R and L<br>Posterior thalamic radiation[a] R and L<br>Sagittal stratum[b] R and L<br>External capsule R and L<br>Fornix (cres) / Stria terminalis R and L |

| | | |
|---|---|---|
| | | Superior longitudinal fasciculus R and L<br>Superior fronto-occipital fasciculus R<br>Uncinate fasciculus R and L<br>Tapetum R and L |
| preserved balance aTBI FA < control FA | 0.07 | |
| preserved balance aTBI MD > control MD | 0.29 | |
| impaired balance aTBI FA < preserved balance aTBI FA | **0.04*** | CC (genu)<br>Anterior corona radiata L |
| impaired balance aTBI MD > preserved balance aTBI MD | **0.03*** | CC (genu)<br>Anterior corona radiata L<br>External capsule L |
| **C) Whole brain correlation contrasts** | | |
| *C1. Vestibular-mediated balance* | | |
| All participants FA to balance (-) | **0.005**** | CC (genu, body, splenium)<br>Fornix (column and body of fornix)<br>Cerebral peduncle R and L<br>Anterior limb of internal capsule R<br>Posterior limb of internal capsule R and L<br>Retrolenticular part of internal capsule R and L<br>Anterior corona radiata R and L<br>Superior corona radiata R and L<br>Posterior corona radiata R and L<br>Posterior thalamic radiation[a] R and L<br>Sagittal stratum[b] R and L<br>External capsule R and L<br>Cingulum (cingulate gyrus) R<br>Fornix (cres) / Stria terminalis R and L<br>Superior longitudinal fasciculus R and L<br>Superior fronto-occipital fasciculus R<br>Uncinate fasciculus R<br>Tapetum R and L |
| All participants MD to balance (+) | **0.02*** | CC (genu, body, splenium)<br>Anterior limb of internal capsule R and L<br>Retrolenticular part of internal capsule R and L<br>Anterior corona radiata R and L<br>Superior corona radiata R and L<br>Posterior corona radiata R and L<br>Posterior thalamic radiation[a] R and L<br>Sagittal stratum[b] R and L<br>External capsule R and L<br>Fornix (cres) / Stria terminalis R and L<br>Superior longitudinal fasciculus R and L<br>Superior fronto-occipital fasciculus R<br>Uncinate fasciculus R<br>Tapetum R and L |
| aTBI FA to balance (-) | **0.04*** | CC (genu, body, splenium)<br>Cerebral peduncle R<br>Posterior limb of internal capsule R<br>Retrolenticular part of internal capsule R<br>Anterior corona radiata R and L<br>Superior corona radiata R<br>Posterior corona radiata R<br>Posterior thalamic radiation[a] R<br>Sagittal stratum[b] R<br>External capsule R<br>Superior longitudinal fasciculus R<br>Tapetum R |
| aTBI MD to balance (+) | **0.04*** | CC (splenium)<br>Retrolenticular part of internal capsule R<br>Superior corona radiata R<br>Posterior corona radiata R and L<br>Posterior thalamic radiation[a] R<br>Sagittal stratum[b] R<br>External capsule R<br>Fornix (cres) / Stria terminalis R<br>Superior longitudinal fasciculus R and L |
| control FA to balance (-) | 0.16 | |
| control MD to balance (+) | 0.28 | |
| VA+ FA to balance (-) | 0.09 | |
| VA+ MD to balance (+) | **0.063** | [#]Posterior thalamic radiation[a] R<br>[#]Sagittal stratum[b] R<br>[#]External capsule R |
| VA- FA to balance (-) | 0.21 | |
| VA- MD to balance (+) | 0.17 | |

| | | |
|---|---|---|
| impaired balance aTBI FA to balance (-) | 0.24 | |
| impaired balance aTBI MD to balance (+) | 0.12 | |
| preserved balance aTBI FA to balance (-) | 0.23 | |
| preserved balance aTBI MD to balance (+) | 0.17 | |
| *C2. Vestibular-perceptual thresholds* | | |
| All participants FA to VPT (-) | 0.10 | |
| All participants MD to VPT (+) | **0.04*** | Sagittal stratum[b] R |
| aTBI FA to VPT (-) | 0.23 | |
| aTBI MD to VPT (+) | 0.10 | |
| control FA to VPT (-) | 0.89 | |
| control MD to VPT (+) | 0.80 | |
| VA+ FA to VPT (-) | 0.20 | |
| VA+ MD to VPT (+) | 0.11 | |
| VA- FA to VPT (-) | 0.57 | |
| VA- MD to VPT (+) | 0.32 | |
| impaired balance aTBI FA to VPT (-) | **0.07** | $Posterior thalamic radiation[a] R<br>$Sagittal stratum[b] R |
| impaired balance aTBI MD to VPT (+) | **0.05*** | Sagittal stratum[b] R |
| preserved balance aTBI FA to VPT (-) | 0.53 | |
| preserved balance aTBI MD to VPT (+) | 0.27 | |
| *C3. Vestibular-ocular reflex thresholds* | | |
| All participants FA to VOR (-) | 0.13 | |
| All participants MD to VOR (+) | 0.13 | |
| aTBI FA to VOR (-) | 0.20 | |
| aTBI MD to VOR (+) | 0.33 | |
| control FA to VOR (-) | 0.64 | |
| control MD to VOR (+) | 0.26 | |
| VA+ FA to VOR (-) | 0.29 | |
| VA+ MD to VOR (+) | 0.25 | |
| VA- FA to VOR (-) | 0.53 | |
| VA- MD to VOR (+) | 0.68 | |
| impaired balance aTBI FA to VOR (-) | **0.02*** | CC (genu, body, splenium)<br>Anterior limb of internal capsule R<br>Retrolenticular part of internal capsule R<br>Anterior corona radiata R<br>Superior corona radiata R<br>Posterior corona radiata R<br>Posterior thalamic radiation[a] R<br>Sagittal stratum[b] R<br>External capsule R<br>Fornix (cres) / Stria terminalis R<br>Superior longitudinal fasciculus R<br>Superior fronto-occipital fasciculus R<br>Uncinate fasciculus R |
| impaired balance aTBI MD to VOR (+) | **0.007**** | Retrolenticular part of internal capsule R<br>Sagittal stratum[b] R<br>External capsule R |
| preserved balance aTBI FA to VOR (-) | 0.44 | |
| preserved balance aTBI MD to VOR (+) | 0.66 | |

Abbreviations: aTBI, acute traumatic brain injury patients; FA, fractional anisotropy; MD, mean diffusivity; VA+, vestibular agnosia patients; VA-, patients without vestibular agnosia; VPT, vestibular-perceptual thresholds; VOR, vestibular-ocular reflex thresholds; +, positive correlation contrast between DTI parameter and behavioural measure; -, negative correlation contrast between DTI parameter and behavioural measure; CC, corpus callosum; R, right; L, left. * $p < 0.05$; ** $p < 0.01$; Posterior thalamic radiation[a], include optic radiation; Sagittal stratum[b], include inferior longitudinal fasciculus and inferior fronto-occipital fasciculus; #, tendency, threshold set at 0.065; $, tendency, threshold set at 0.075.
